# New Functions and Subcellular Localization Patterns of c-di-GMP Components (GGDEF Domain Proteins) in *B. subtilis*

**DOI:** 10.3389/fmicb.2017.00794

**Published:** 2017-05-09

**Authors:** Patricia Bedrunka, Peter L. Graumann

**Affiliations:** LOEWE SYNMIKRO, LOEWE Center for Synthetic Microbiology and Department of Chemistry, Philipps University Marburg, Hans-Meerwein StrasseMarburg, Germany

**Keywords:** *Bacillus subtilis*, Biofilm formation, c-di-GMP signaling, exopolymeric substances, signal transduction, protein dynamics

## Abstract

The universal and pleiotropic cyclic dinucleotide second messenger c-di-GMP is most prominently known to inversely regulate planktonic and sessile lifestyles of Gram-negative species. In the Gram-positive model organism *Bacillus subtilis*, intracellular c-di-GMP levels are modulated by a concise set of three diguanylate cylases (DgcK, DgcP, DgcW) and one phosphodiesterase (PdeH). Two recent studies have reported the negative influence of the c-di-GMP receptor DgrA (PilZ domain protein) on swarming motility indicating a conserved role of this second messenger across the bacterial domain. However, it has been suggested that the degenerated GGDEF protein YdaK and the inactive EAL domain protein YkuI may also function as c-di-GMP receptors regulating potentially other processes than motility. Here we describe a novel c-di-GMP dependent signaling network in *B. subtilis* regulating the production of an unknown exopolysaccharide (EPS) that leads to strongly altered colony morphologies upon overproduction. The network consists of the c-di-GMP receptor YdaK and the c-di-GMP synthetase DgcK. Both proteins establish a spatially close signal-effector cluster at the membrane. The cytoplasmic DgcP synthetase can complement for DgcK only upon overproduction, while the third c-di-GMP synthetase, DgcW, of *B. subtilis* is not part of the signaling pathway. Removal of the regulatory EAL domain from DgcW reveals a distinct function in biofilm formation. Therefore, our study is compatible with the “local pool signaling” hypothesis, but shows that in case of the *yda* operon, this can easily be overcome by overproduction of non-cognate DGCs, indicating that global pools can also confer signals to regulatory circuits in a Gram-positive bacterium.

## Introduction

Bacteria utilize a multitude of regulatory processes to ensure the adaptation to environmental changes for the sake of growth- and survival optimization. Upon detection of diverse primary signals, transduction of these external stimuli into a cellular response can be ubiquitously realized by the production of purine nucleotide derivatives (Gomelsky, 2011; Hengge et al., 2016). The dynamic synthesis- and degradation mechanisms of these so called second messengers have an enormous impact on corresponding cellular downstream effects as they determine and modulate the cellular levels and therefore, also (at least to some extent) the probability of interaction between second messengers and their specific effector molecules (effector proteins and/or riboswitches, reviewed in Ryan et al., 2012).

Bis-(3′–5′)-cyclic dimeric guanosine monophosphate (c-di-GMP) is a well-established purine second messenger regulating most notably bacterial lifestyle orchestration. The consensus of numerous studies implies that an increase in c-di-GMP production correlates with a sessile lifestyle [biofilm (BF) formation], whereas low c-di-GMP levels favor planktonic cell behavior. Specific diguanylate cyclases (DGCs) harbor conserved GGDEF domains and synthesize c-di-GMP from two molecules of guanosine-5′-triphosphate (GTP), whereas specific phosphodiesterases (PDEs, containing either EAL or HD-GYP domains) mediate its degradation into the linear dinucleotide 5′-phosphoguanylyl-(3′,5′)-guanosine (pGpG) and/or GMP (Romling et al., 2013; Jenal et al., 2017). Interestingly, these characteristic domains are frequently combined with diverse N-terminal soluble and/or membrane-integrated domains which are primarily utilized for sensory purposes in order to modulate DGC and PDE activities respectively (Plate and Marletta, 2012; Zahringer et al., 2013).

Another fundamental regulatory process of intracellular c-di-GMP homeostasis is the allosteric product inhibition of DGC activity (Christen et al., 2006; De et al., 2008; Schirmer and Jenal, 2009). This is achieved by the interaction of c-di-GMP with conserved auto-inhibitory I-site motifs (primary and secondary) in order to prevent over-consumption and excessive production of the substrate and the product respectively. Very recently, it was demonstrated that I-sites do not only contribute to the maintenance of c-di-GMP homeostasis as negative regulatory elements, but can also positively regulate the physical interaction of an active DGC with its specific c-di-GMP receptor (Dahlstrom et al., 2016). I-site motifs can furthermore function as c-di-GMP “receptor sites” (activation motifs) of enzymatically inactive (degenerated) GGDEF domains to drive exopolysaccharide (EPS) synthesis for example (Chen et al., 2014). By limiting the total amount of c-di-GMP available and additionally providing an interaction platform for corresponding effector molecules and interaction partners respectively, I-site motifs of active and inactive GGDEF domains are thus able to modulate diverse levels of signal specificity.

Recently, we have proven the requirement of the potential c-di-GMP effector protein YdaK, a degenerated GGDEF-TM protein, for the synthesis of an extracellular matrix component generated by the products of the *yda*(*J*)*KLMN* (*ydaJ*-*N*) operon in *Bacillus subtilis* (Nicolas et al., 2012; Bedrunka and Graumann, 2017). The unknown EPS strongly affects Congo Red (CR) binding and the characteristic morphology of *B. subtilis* macro colonies grown on BF-promoting medium, for example. Enhanced CR-binding can be likewise visualized in the absence of *epsH* belonging to the *epsA*-*O* cluster, which implies the production of an alternative EPS in case of *ydaJ*-*N* overexpression. Whether *ydaJ*-*N* overexpression has an effect on the expression of other matrix gene operons such as *epsA*-*O* and *tapA*-*sipW*-*tasA* remains to be clarified. In contrast to the *epsA*-*O* operon, which is essential for development of complex colony and pellicle BFs (Kearns et al., 2005), deletions targeting the *ydaJ*-*N* operon have no influence on the establishment of such BFs (Gao et al., 2013; Bedrunka and Graumann, 2017). Importantly, the influence of *ydaJ*-*N* on colony BF architecture can be recognized only upon overexpression (Figure S1). Its potential significance on BF formation therefore requires further investigations using different experimental systems and conditions.

However, our previous findings provide a new tool to study the effect of c-di-GMP in *B. subtilis* with respect to EPS production via YdaK. For a while now, degenerated GGDEF domains have been known to function as positive regulators of EPS production most likely via their conserved I-site motifs (Liang, 2015), a mechanism that has been also proposed for YdaK. The TM-protein is not able to synthesize c-di-GMP, still it can bind the second messenger *in vitro* via its soluble degenerated GGDEF domain (Gao et al., 2013).

In this study, we wanted to further investigate the potential c-di-GMP/I-site dependent activation of EPS synthesis in *B. subtilis* and were especially interested whether a physiological relation between YdaK and *B. subtilis* DGCs does exist and whether this EPS promoting putative c-di-GMP effector can be genetically linked to the activity of one specific DGC.

**Figure 1:**
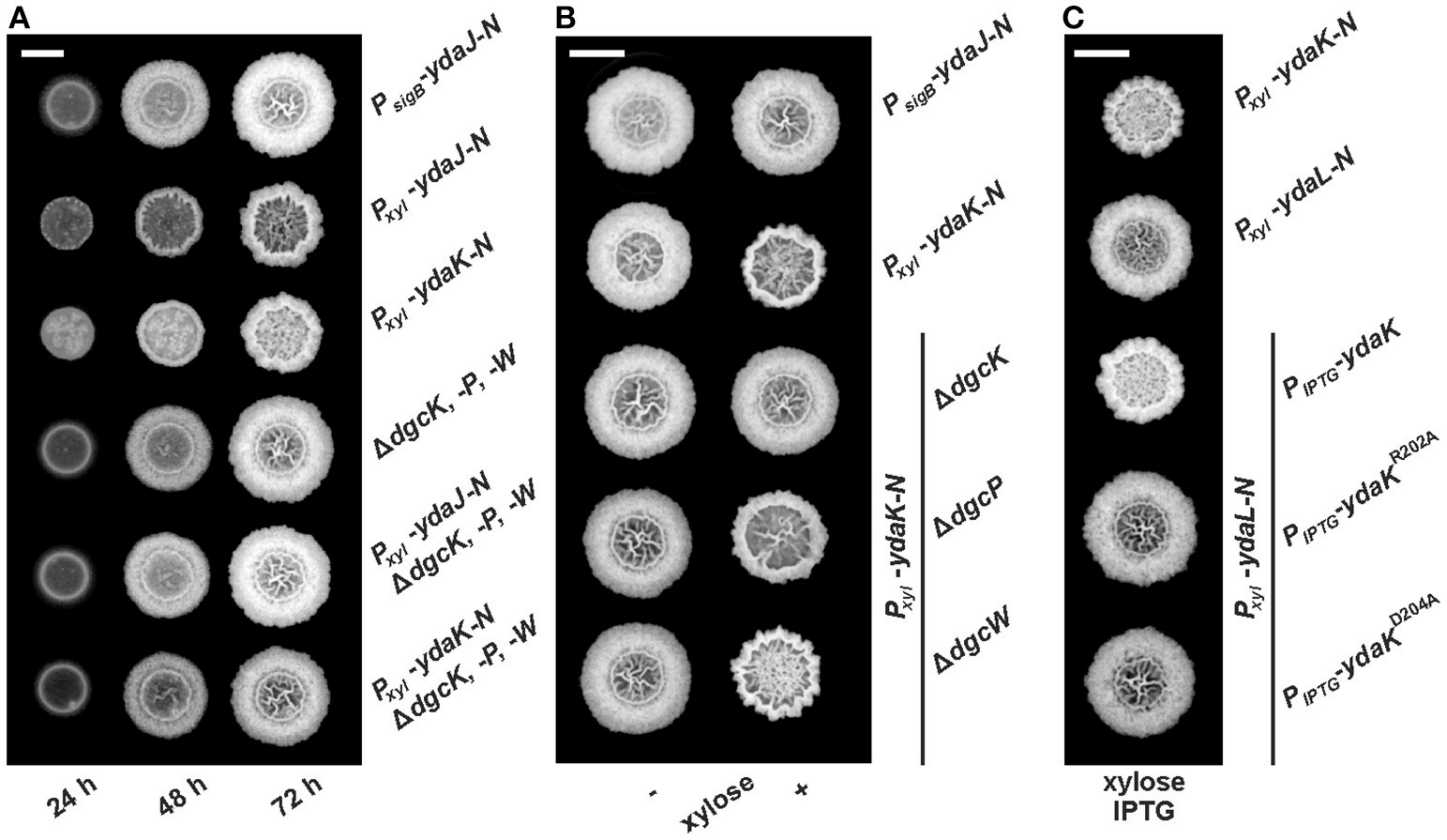
**Combinatorial deletions of ***dgc*** genes and particularly inactivation of ***dgcK*** and disruption of the putative YdaK I-site motif RxxD lead to an inhibition of Yda(J)KLMN-mediated EPS production in ***B. subtilis***. (A)** Top view of *B. subtilis* macro colony morphology and expansion on biofilm promoting medium (MSgg, Branda et al., 2001) supplemented with 0.1% (v/v) xylose, 40 μg/ml Congo Red (CR) and 20 μg/ml Coomassie Brilliant Blue (CB) at different timepoints for WT NCIB3610 (*P*_*sigB*_-*ydaJ*-*N*), NCIB3610-PB53 (*P*_*xyl*_-*ydaJ*-*N*), NCIB3610-PB55 (*P*_*xyl*_-*ydaK*-*N*), DS1809 (Δ*dgcK*, -*P*, -*W*), DS1809-PB53 (Δ*dgcK*, -*P*, -*W, P*_*xyl*_-*ydaJ*-*N*), and DS1809-PB55 (Δ*dgcK*, -*P*, -*W, P*_*xyl*_-*ydaK*-*N*) at 25°C. Reduced colony expansion and altered wrinkle patterns (*hyper*-*wrinkles*) indicate EPS production by the products of the *yda* operon. **(B)** *B. subtilis* biofilm morphology on MSgg (+ CR, CB) solid medium 72 h post-inoculation in the absence and presence of 0.1% (v/v) xylose for wild type strain NCIB3610 (*P*_*sigB*_-*ydaJ*-*N*), the overexpression strain NCIB3610-PB55 (*P*_*xyl*_-*ydaK*-*N*) and combined overexpression and deletion mutants: DS9305-PB55 (Δ*dgcK*; *P*_*xyl*_-*ydaK*-*N*), DS9537-PB55 (Δ*dgcP*; *P*_*xyl*_-*ydaK*-*N*), and DS9883-PB55 (Δ*dgcW*; *P*_*xyl*_-*ydaK*-*N*). **(C)** Colony morphology of the overproduction mutant strains NCIB3610-PB55 (*P*_*xyl*_-*ydaK*-*N*) and NCIB3610-PB56 (*P*_*xyl*_-*ydaL*-*N*) and the complementation strains NCIB3610-PB56-XG003 (*P*_*xyl*_-*ydaL*-*N*; *amyE*::*P*_*IPTG*_-*ydaK*), NCIB3610-PB56-PB80 (*P*_*xyl*_-*ydaL*-*N, amyE*::*P*_*IPTG*_-*ydaK*^R202A^), NCIB3610-PB56-PB81 (*P*_*xyl*_-*ydaL*-*N, amyE*::*P*_*IPTG*_-*ydaK*^D205A^) grown at 25°C on MSgg (+CR, CB) solid medium with 0.1% (v/v) xylose and 1 mM IPTG after 72 h. Bars correspond to 5 mm.

Three different enzymes are capable of c-di-GMP synthesis in *B. subtilis*: the two GGDEF domain proteins DgcK and DgcP (formerly YhcK and YtrP) and the GGDEF-EAL domain protein DgcW (YkoW). The current knowledge on the cellular roles of c-di-GMP synthesizing enzymes in *B. subtilis* is limited to motility control, mediated by the interaction between DgrA (formerly YpfA, PilZ- domain protein) and the flagellar component MotA upon elevated intracellular c-di-GMP levels. However, the regulatory modes and physiological functions of these three DGCs with respect to EPS production/ BF formation and motility inhibition respectively remained unknown (Chen et al., 2012; Gao et al., 2013). Inactivation of *dgc* genes (*dgcK, dgcP, dgcW*) in undomesticated *B. subtilis* strains, individually or in combination, results in no detectable phenotypes with respect to BF formation and motility (Figures 1A,B; Chen et al., 2012; Gao et al., 2013). However, overproduction of the GGDEF domain proteins DgcK and DgcP, as well as an overproduction of a DgcW variant lacking the adjacent EAL domain, respectively, causes a transient inhibition of swarming motility (Gao et al., 2013). Under these circumstances, or upon deletion of the sole PDE gene *pdeH* (formerly *yuxH*), elevated intracellular c-di-GMP concentrations could be detected resulting in premature motility cessation via the “high affinity” c-di-GMP receptor DgrA [dissociation constant (K_D_) 11 nM] but notably, in no observable alterations concerning BF formation (Gao et al., 2013).

To approach the cellular functions of DGCs in *B. subtilis* with respect to EPS production/colony BF formation, we generated diverse combinations of overexpression and deletion mutants. By examining their behavior toward BF formation, we are able to provide genetic and cell biological evidence for the existence of novel and distinct functions for DgcK, DgcP, and DgcW. In order to extend our understanding of c-di-GMP signaling components in *B. subtilis*, a comparative fluorescence microscopy analysis of YdaK and its putative c-di-GMP delivering synthetases was carried out. Importantly, we show that both YdaK and DgcK fluorescent fusions form subcellular clusters and co-localize to the same cellular positions. Additionally, they exhibit similar dynamic behavior suggesting a physiological connection between YdaK and DgcK, as already implicated by our phenotypical analysis.

## Results

### Cell biological evidence for an implication of c-di-GMP in BF formation in *B. subtilis*

The “low affinity” c-di-GMP receptor YdaK (K_D_ 1 μM) does not serve as an effector protein to modulate swarming motility directly (Gao et al., 2013), but instead affects BF formation by positively regulating the production of an unknown EPS synthesized by the products of the *ydaJ*-*N* operon (Bedrunka and Graumann, 2017).

We wanted to investigate the potential involvement of DGCs and thus c-di-GMP in EPS production via induction of YdaK. Deletion and overexpression of *ydaK* alone have not revealed any phenotypic effects so far. Therefore, we initially introduced two expression constructs pSG1164-PB53 (*P*_*xyl*_-*ydaJ*-*N*) and pSG1164-PB55 (*P*_*xyl*_-*ydaK*-*N*), steering the expression of the transcriptional units *ydaJ*-*N* and *ydaK*-*N* respectively, into the genome of wild type *B. subtilis* NCIB3610, and into that of a *dgc* triple mutant strain lacking the native c-di-GMP-synthesizing components (strain DS1809, Δ*dgcK* Δ*dgcW dgcP*::*tet*, Figure 1A).

As shown in an earlier study (Bedrunka and Graumann, 2017), induction of *ydaJ*-*N* (strain NCIB3610-PB53, *P*_*xyl*_-*ydaJ*-*N*) and overexpression of *ydaK*-*N* (strain NCIB3610-PB55, *P*_*xyl*_-*ydaK*-*N*) respectively, lead to strongly altered and wrinkled colony morphologies and inhibited surface spreading behavior (Figure S1 and Figure 1A, second and third row, respectively) of *B. subtilis* on solid BF medium (Branda et al., 2001). These changes are indicative of EPS production as demonstrated for different bacterial species (Serra et al., 2013).

Essentially, inactivation of all three *dgc* genes prevents the synthesis of this unknown EPS in both induction strains (Figure 1A, fifth row: strain DS1809-PB53, Δ*dgcK*, -*P*, -*W, P*_*xyl*_-*ydaJ*-*N*; sixth row: strain DS1809-PB55, Δ*dgcK*, -*P*, -*W, P*_*xyl*_-*ydaK*-*N*). The morphology of these resembled that of the wild type supporting the c-di-GMP dependence of the *ydaJ-N* related EPS machinery (Figure 1A, first row).

We proceeded to test the effect of *ydaK*-*N* induction (construct pSG1164-PB55, *P*_*xyl*_-*ydaK*-*N*) on BF-promoting medium in single *dgc* gene mutant backgrounds (Figure 1B). Importantly, in strains in which the endogenous locus *dgcK* was deleted (strain DS9305-PB55, Δ*dgcK, P*_*xyl*_-*ydaK*-*N*, Figure 1B, third row) production of EPS by YdaK-N was completely abolished despite the presence of xylose, as respective colony architectures resembled wild type appearance (NCIB3610, *P*_*sigB*_-*ydaJ*-*N*, Figure 1B, first row). In contrast, disruption of *dgcP* did not impair EPS production via YdaK-N, reflected by the altered colony morphology in the case *ydaK*-*N* induction (strain DS9537-PB55, Δ*dgcP, P*_*xyl*_-*ydaK*-*N*, Figure 1B, right column, forth row), compared to the *dgcK* background deletion strain (Figure 1B, right column, third row). Similarly, deletion of *dgcW* also resulted in altered BF formation upon induction of *ydaK*-*N* (strain DS9883-PB55, Δ*dgcW, P*_*xyl*_-*ydaK*-*N*, Figure 1B right column, fifth row) implying that EPS production via YdaK-N is independent of DgcP and DgcW under our experimental conditions, but dependent of DgcK.

Thus, our experiments demonstrate that activation of EPS production via the degenerated GGDEF domain protein YdaK relies on the integrity of *dgc* genes and furthermore indicate that YdaK is activated via DgcK under BF-promoting conditions, which results in activation of EPS production in a c-di-GMP dependent manner. In order to further support the hypothesis of YdaK activation via c-di-GMP, we performed site directed replacement mutagenesis of conserved I-site (inhibitory site) residues in YdaK proposed to be involved in c-di-GMP binding (Gao et al., 2013, Figure 1C). Based on sequence analysis, YdaK likely has a primary and secondary inhibitory site (Gao et al., 2013). The putative secondary I-site motif RxxR is found at residues R157 to R160, whereas the putative primary I-site motif RxxD (R202 to D205) locates five residues upstream of the degenerated active site motif SDERI (conserved motif GGDEF). To test the activity of the primary I-site variants, YdaK^R202A^ and YdaK^D205A^ (pXG003-PB80 & pXG003-PB81, respectively) were introduced at the ectopic *amyE* locus of strains that harbored a xylose-dependent promoter driving *ydaLMN* expression (pSG1164-PB56; Figure 1C). Induction of *ydaLMN* alone (strain NCIB3610-PB56, *P*_*xyl*_-*ydaL*-*N*, Figure 1C, second row) did not result in EPS production as reflected by unaltered colony morphology of the correponding strain compared to *ydaK*-*N* overexpression (strain NCIB3610-PB55, *P*_*xyl*_-*ydaK*-*N*; Figure 1C, first row) in agreement with a previous study (Bedrunka and Graumann, 2017). Complementation with a wild type copy of *ydaK in trans* restored EPS production upon xylose and IPTG addition (strain NCIB3610-PB56-XG003, *P*_*xyl*_-*ydaLMN, amyE*::*P*_*IPTG*_-*ydaK*, Figure 1C, third row). However, when strain NCIB3610-PB56 (*P*_*xyl*_-*ydaL*-*N*) was complemented with *ydaK* alleles encoding the R202A (NCIB3610-PB56-PB80, *P*_*xyl*_-*ydaLMN, amyE*::*P*_*IPTG*_-*ydaK*^R202A^, Figure 1C, forth row) or D205A point mutants (NCIB3610-PB56-PB81, *P*_*xyl*_-*ydaLMN, amyE*::*P*_*IPTG*_-*ydaK*^D205A^, Figure 1C, fifth row) respectively, EPS production was abolished. These observations suggest that an intact primary I-site motif of YdaK is required for c-di-GMP binding and thus YdaK activity.

### DgcK, DgcP, and truncated DgcW (“ΔEAL”) can activate EPS production in *B. subtilis* via overproduced YdaK

**Figure 2:**
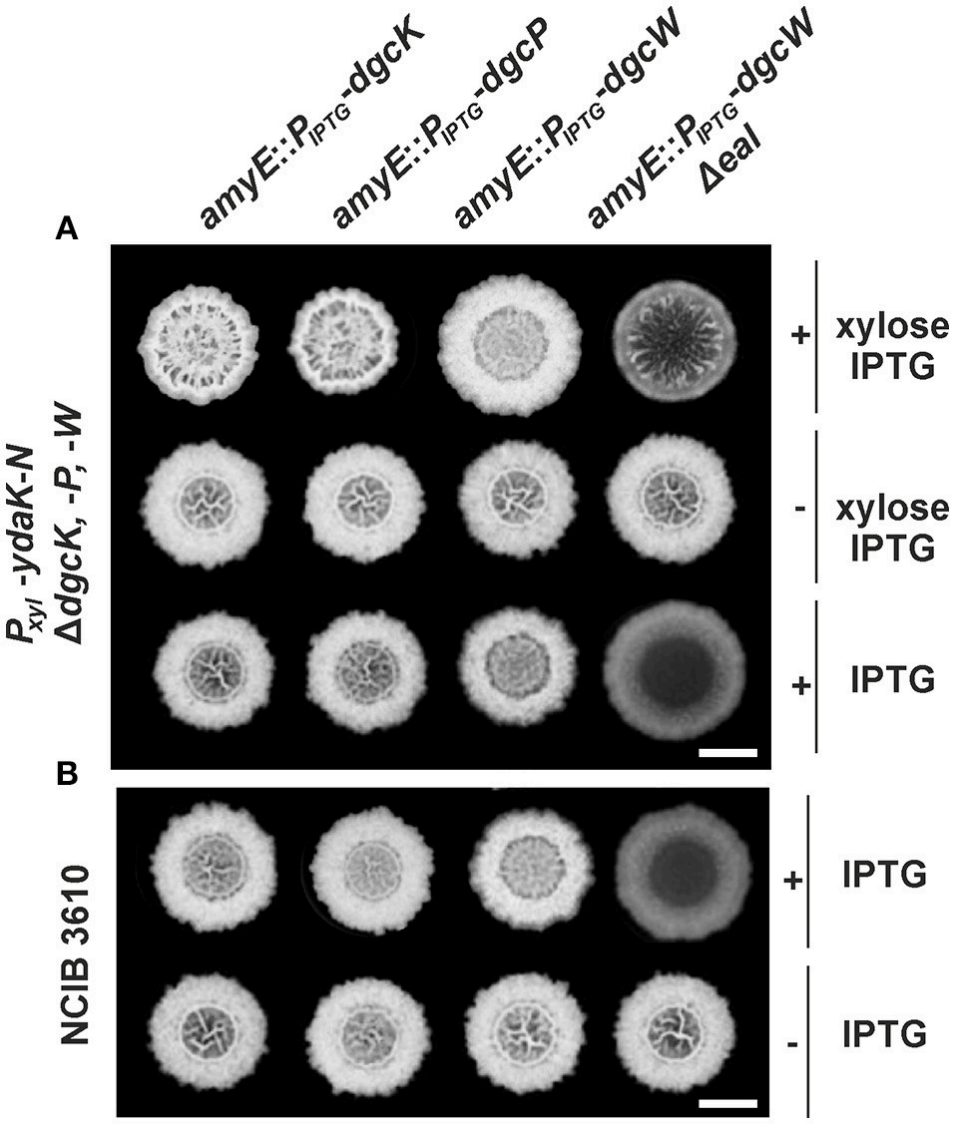
**Altered biofilm formation due to ***ydaK-N*** overexpression can be restored in a ***dgc*** triple mutant by providing ***dgcK***, ***dgcP***, and ***dgcW***Δ***eal in trans***. (A)** Colony development on MSgg medium supplemented with CR 40 μg/ml, CB 20 μg/ml in the presence or absence of 0.1% (v/v) xylose and/ or 1 mM IPTG, respectively, 72 h after incubation at 25°C by strains: DS1809-PB55-XG004 (Δ*dgcK*, -*P*, -*W, P*_*xyl*_-*ydaK*-*N, amyE*::*P*_*IPTG*_-*dgcK*), DS1809-PB55-XG002 (Δ*dgcK*, -*P*, -*W, P*_*xyl*_-*ydaK*-*N, amyE*::*P*_*IPTG*_-*dgcP*), DS1809-PB55-XG001 (Δ*dgcK*, -*P*, -*W, P*_*xyl*_-*ydaK*-*N, amyE*::*P*_*IPTG*_-*dgcW*), DS1809-PB55-XG086 (Δ*dgcK*, -*P*, -*W, P*_*xyl*_-*ydaK*-*N, amyE*::*P*_*IPTG*_- *dgcW*Δ*eal*). Scale bar: 5 mm. **(B)** Biofilm colony morphology of *B. subtilis* NCIB3610 strains individually overexpressing *dgcK* (strain NCIB3610-XG004, *amyE*::*P*_*IPTG*_-*dgcK*), *dgcP* (NCIB3610-XG002, *amyE*::*P*_*IPTG*_-*dgcP*), *dgcW* (NCIB3610-XG001, *amyE*::*P*_*IPTG*_-*dgcW*), and *dgcW*Δ*eal* (NCIB3610-XG086, *amyE*::*P*_*IPTG*_-*dgcW*Δ*eal*) from the ectopic amylase locus. Scale bars: 5 mm.

Given that EPS production mediated by YdaK-N is suppressed in the absence of all three *dgc* genes and specifically upon disruption of *dgcK* (Figures 1A,B), we were interested whether the loss of the *ydaK*-*N* expression phenotype in a *dgc* triple mutant can be complemented only with *dgcK* or whether additional *dgc* genes (*dgcP, dgcW*) can positively influence the production of the unknown EPS, e.g., when they are overexpressed. Therefore, we introduced each *dgc* gene individually at the ectopic *amyE* locus under the control of an IPTG-inducible promoter, into the genome of the *dgc* triple mutant, which induces *ydaK*-*N* upon xylose addition, and tested BF formation in the presence or absence of xylose and IPTG, respectively (Figure 2A). As expected, the overexpression of *dgcK* restored modified BF formation upon addition of xylose and IPTG (Figure 2A, first column, first row, strain: DS1809-PB55-XG004, Δ*dgcK*, -*P*, -*W, P*_*xyl*_-*ydaK*-*N, amyE*::*P*_*IPTG*_-*dgcK*) as observed in wild type backgrounds (Figure 1B, second column, second row, strain NCIB3610-PB55, *P*_*xyl*_-*ydaK*-*N*). Interestingly, also an overexpression of *dgcP* restored hyper-wrinkle formation (Figure 2A, second column, first row, strain: DS1809-PB55-XG002, Δ*dgcK*, -*P*, -*W, P*_*xyl*_-*ydaK*-*N, amyE*::*P*_*IPTG*_-*dgcP*).

In contrast to *dgcK* and *dgcP* (encoding GGDEF domain proteins), reintroduction of full length *dgcW* (coding for a GGDEF-EAL domain tandem protein) at the *amyE* site (Figure 2A, third column, first row, strain: DS1809-PB55-XG001, Δ*dgcK*, -*P*, -*W, P*_*xyl*_-*ydaK*-*N, amyE*::*P*_*IPTG*_-*dgcW*) did not restore altered biofilm morphology of strain NCIB3610-PB55 (*P*_*xyl*_-*ydaK*-*N*). However, colonies grown in the presence of IPTG/xylose (Figure 2A, third column, first row) and IPTG (Figure 2A, third column, third row) respectively, exhibited a distinct phenotype compared to the wild type (Figure 1A, first column, first row). Overexpression of *dgcW* resulted in a visible reduction of thick wrinkle structures and in a loss of the associated central ring that usually marks the initial inoculation area, in a *dgc* triple mutant overexpressing *ydaK*-*N* (Figure 2A, third column, first row) and also in wild type background (Figure 2B, third column, first row).

This rather modest but reproducible BF-inhibiting phenotype became more severe when a DgcW variant was overproduced lacking the C-terminal EAL domain (Figure 2A, forth column, third row; Figure 2B, forth column, first row). Overexpression of *dgcW*-Δ*eal* caused an entire loss of wrinkle formation in the center of the macro-colony in both genomic backgrounds, in DS1809-PB55 (Figure 2A, forth column, third row, strain DS1809-PB55-XG086, Δ*dgcK*, -*P*, -*W, P*_*xyl*_-*ydaK*-*N, amyE*::*P*_*IPTG*_-*dgcW*Δ*eal*) and in NCIB3610 (Figure 2B, forth column, first row, strain NCIB3610-XG086, *amyE*::*P*_*IPTG*_-*dgcW*Δ*eal*). Furthermore, we found that induction of *ydaK*-*N* accompanied by an overexpression of *dgcW*-Δ*eal* lead to enhanced wrinkle formation and altered BF formation (Figure 2A, forth column, first row, strain DS1809-PB55-XG086, Δ*dgcK*, -*P*, -*W, P*_*xyl*_-*ydaK*-*N, amyE*::*P*_*IPTG*_-*dgcW*Δ*eal*). This suggests that a truncated version of DgcW is able to provide c-di-GMP to activate EPS production via YdaK.

In summary, our complementation analysis reveals that both DgcK and DgcP support EPS production when being overproduced, whereas DgcW is only able to do so upon deletion of the GGDEF-adjacent EAL domain. Therefore, we suggest that the activity of the EAL domain (potentially c-di-GMP hydrolysis) of DgcW masks the enzymatic activity of its neighboring (upstream) GGDEF domain (c-di-GMP synthesis). Because the overproduction of DgcW-ΔEAL by itself had a strong effect on BF morphology, our findings suggest that DgcW affects a pathway different from that of the *yda* operon, and that therefore, at least two independent c-di-GMP processes occur during BF formation in case of *ydaK*-*N* and *dgcW*Δ*eal* overexpression.

### Subcellular localization and dynamics of the c-di-GMP receptor YdaK and the DGCs DgcK, and DgcP in *B. subtilis*

Several DGCs in different bacterial species have been reported to occur in complex with their effector proteins/targets in order to maintain signal specificity within certain signaling cascades (Lindenberg et al., 2013; Dahlstrom et al., 2015). Additionally, it has been suggested that differential subcellular localization patterns of DGCs may affect their function and thus the interplay with its corresponding effectors (Merritt et al., 2010; Zhu et al., 2016). This prompted us to perform comparative localization studies of the c-di-GMP receptor YdaK and of the corresponding DGCs (DgcK and DgcP) that were able to restore EPS production mediated by YdaK-N in a *dgc* triple mutant (Figure 2A).

In a recent study, we investigated the subcellular localization of YdaK and of its potential downstream targets, the putative glycosyltransferases YdaM and YdaN, by fluorescence microscopy (Bedrunka and Graumann, 2017). YdaK-, YdaM-, and YdaN-mV-YFP fusions (mV: monomeric Venus) expressed from their original promoter and at their native site on the chromosome, formed static subcellular clusters (usually one or two single foci per cell) at the membrane, predominantly at the septa and/or at cell poles in exponentially growing *B. subtilis* cells. YdaM/YdaK and YdaM/YdaN fluorescent fusions co-localized to the same cellular positions supporting the idea that specific protein localization at the cell membrane might be necessary to facilitate protein-protein interactions and EPS production.

**Figure 3:**
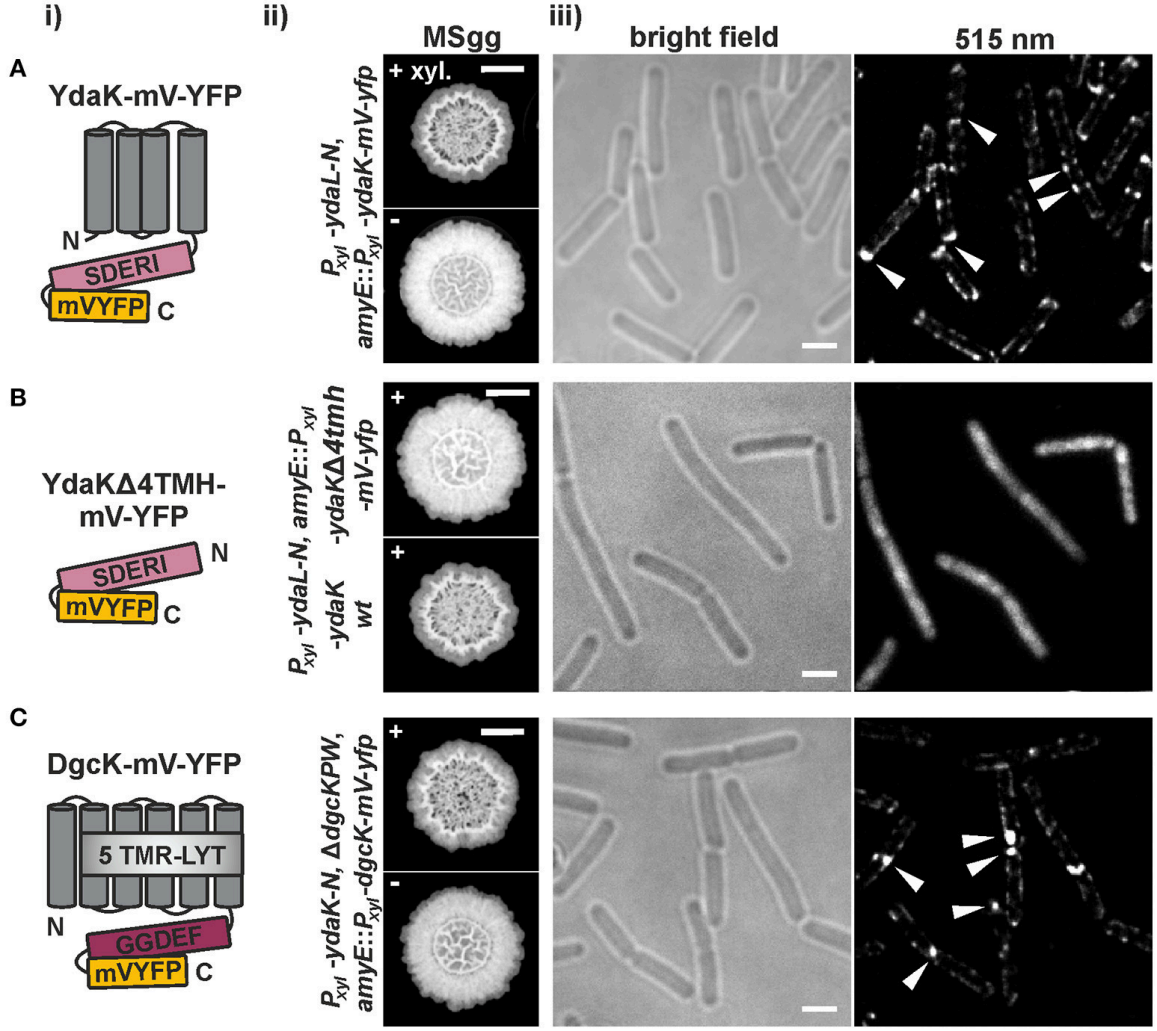
**Functional translational mV-YFP-fusions of the c-di-GMP receptor YdaK and of the synthase DgcK form subcellular assemblies at the cell poles and septa of exponentially growing ***B. subtilis*** NCIB3610 (i)** Schematic representation of **(A)** YdaK-mV-YFP-, **(B)** YdaKΔ4TMH-mV-YFP- and of **(C)** DgcK-mV-YFP- domain organization and topology (predicted by SMART). Gray, TM helices; light gray, predicted TM-receptor domain 5TMR–LYT; purple, GGDEF domain; light purple, GGDEF domain harboring the degenerated active site motif SDERI; yellow, C-terminal mV-YFP. **(ii)** Verification of functionality of **(A)** YdaK-mV-YFP (strain: NCIB3610-PB56-PB57; *P*_*xyl*_-*ydaL*-*N, amyE*::*P*_*xyl*_-*ydaK*-*mV-yfp*) and of **(C)** DgcK-mV-YFP (strain: DS1809-PB55-PB90; Δ*dgcK*, -*P*, -*W, P*_*xyl*_-*ydaK*-*N, amyE*::*P*_*xyl*_-*dgcK*-*mV*-*yfp*). **(B)** Colony morphology of strain NCIB3610-PB56-PB100 (upper panel, *P*_*xyl*_-*ydaL*-*N, amyE*::*P*_*xyl*_-*ydaK*Δ*4tmh*-*mV-yfp*) and NCIB3610-PB56-PB16 (lower panel, *P*_*xyl*_-*ydaL*-*N, amyE*::*P*_*xyl*_-*ydaK*) in the presence of 0.1% (v/v) xylose. Altered colony morphology in the presence of xylose reflects EPS production by the products of the *yda* operon and functionality of the corresponding fusion proteins. Unaltered colony morphology of strain NCIB3610-PB56-PB100 in contrast to strain NCIB3610-PB56-PB16 (lower panel, *P*_*xyl*_-*ydaL*-*N, amyE*::*P*_*xyl*_-*ydaK*) reflects inability of YdaKΔ4TMH-mV-YFP to stimulate EPS production. Scale bars: 5 mm. **(iii)** Mid-exponential-phase *B. subtilis* NCIB3610 cells expressing **(A)** *ydaK*-*mV*-*yfp* (strain: NCIB3610-PB56-PB57; *P*_*xyl*_-*ydaL*-*N, amyE*::*P*_*xyl*_-*ydaK*-*mV-yfp*), **(B)** *ydaK*Δ*4tmh*-*mV-yfp* (strain NCIB3610-PB56-PB100; *P*_*xyl*_-*ydaL*-*N, amyE*::*P*_*xyl*_-*ydaK*Δ*4tmh*-*mV-yfp*) and **(C)** *dgcK*-*mV*-*yfp* (strain NCIB3610-PB90, *amyE*::*P*_*xyl*_-*dgcK*-*mV-yfp*) from the amylase locus, 45 min after induction with 0.1% xylose. Bars: 2 μm. White triangles indicate subcellular clustering of YdaK-mV-YFP and DgcK-mV-YFP respectively.

Functional C-terminal mV-YFP fusions of full-length YdaK induced from the ectopic *amyE* site were able to support EPS activation in a mutant of undomesticated *B. subtilis* that induced *ydaL*-*N* upon xylose addition (strain NCIB3610-PB56-PB57, *P*_*xyl*_-*ydaL*-*N, amyE*::*P*_*xyl*_-*ydaK*-*mV-yfp*, Figure S2) as depicted in Figure 3Aii. Ectopically induced YdaK-mV-YFP fusions primarily localized at the peripheries of cells as double foci at mid cell and/ or at the cell poles revealing high signal intensities but also as lateral patches with rather low signal intensities (Figure 3Aiii). As an additional control, we included a C-terminal fluorescent fusion of YdaK lacking its 4 predicted TMHs (*transmembrane helices*, Figure 3Bi) in this study.

The corresponding construct was introduced into the genome of NCIB3610-PB56 (resulting strain: NCIB3610-PB56-PB100, *P*_*xyl*_-*ydaL*-*N, amyE*::*P*_*xyl*_-*ydaK*Δ*4tmh*-*mV-yfp*; upper panel in Figure 3Bii, Figure S2). In contrast to YdaK-mV-YFP full length fusions (Figure 3A) and wild type YdaK (strain NCIB3610-PB55-PB16, *P*_*xyl*_-*ydaL*-*N, amyE*::*P*_*xyl*_-*ydaK*; lower panel in Figure 3Bii), the truncated variants YdaKΔ4TMH-mV-YFP failed to restore altered BF formation upon overproduction resulting in unaffected colony morphologies. This is most likely due to the fact that removal of TMHs results in a cytoplasmic distribution of these fusion proteins (Figure 3Biii), which is not due to degradation of the fusions as verified by Western blots of cell extracts using an antibody against GFP (Figure S2). Thus, YdaK must localize in a complex with its downstream effector proteins YdaLMN at its native membrane position in order to activate EPS production at specific sites of the bacterial cell membrane, which we have already hypothesized earlier.

In view of these finding, we wondered whether DgcK, the specific c-di-GMP delivering DGC for YdaK (Figure 1B), would resemble YdaK localization (Figure 3C). Initially, we have examined the subcellular localization of C-terminal DgcK-mV-YFP produced from the original locus in *B. subtilis* NCIB3610 cells (strain NCIB3610-PB01, *P*_*dgcK*_*-dgcK*-*mV*-*yfp*). Fusion proteins were hardly detectable suggesting low expression levels of *dgcK*-*mV*-*yfp* under our experimental conditions. Therefore, we visualized the subcellular localization of N- and C-terminal mV-YFP fusions of DgcK originated from the ectopic *amyE* locus upon xylose addition (strain NCIB3610-PB90, *amyE*::*P*_*xyl*_-*dgcK*-*mV-yfp*). Only the translational C-terminal DgcK-mV-YFP proved to be functional (Figure 3Cii, Figure S3). The phenotypes of the *dgc* triple mutant strains, inducing *ydaK*-*N* from the original locus and *dgcK*-*mV*-*yfp* from the *amyE* locus (DS1809-PB55-PB90, Δ*dgcK*, -*P*, -*W, P*_*xyl*_-*ydaK*-*N, amyE*::*P*_*xyl*_-*dgcK*-*mV*-*yfp*), were similar to those of the Δ*dgcK*, -*P*, -*W* strains carrying the *P*_*xyl*_-*ydaK*-*N* construct and the wild-type *dgcK* allele (DS1809-PB55-PB90, Δ*dgcK*, -*P*, -*W, P*_*xyl*_-*ydaK*-*N, amyE*::*P*_*IPTG*_-*dgcK*) in that both strains had altered BF morphologies and were comprised in colony spreading behavior (compare Figure 2A, first column and Figure 3Cii, first row). In contrast to this, overproduction of the fluorescent protein mV-YFP alone from the endogenous locus and induction of *ydaK*-*N* in a triple DGC mutant did not result in EPS production (DS1809-PB55-1193NLMV, Δ*dgcK*, -*P*, -*W, P*_*xyl*_-*ydaK*-*N, amyE*::*P*_*xyl*_-*mV*-*yfp*).

**Figure 4:**
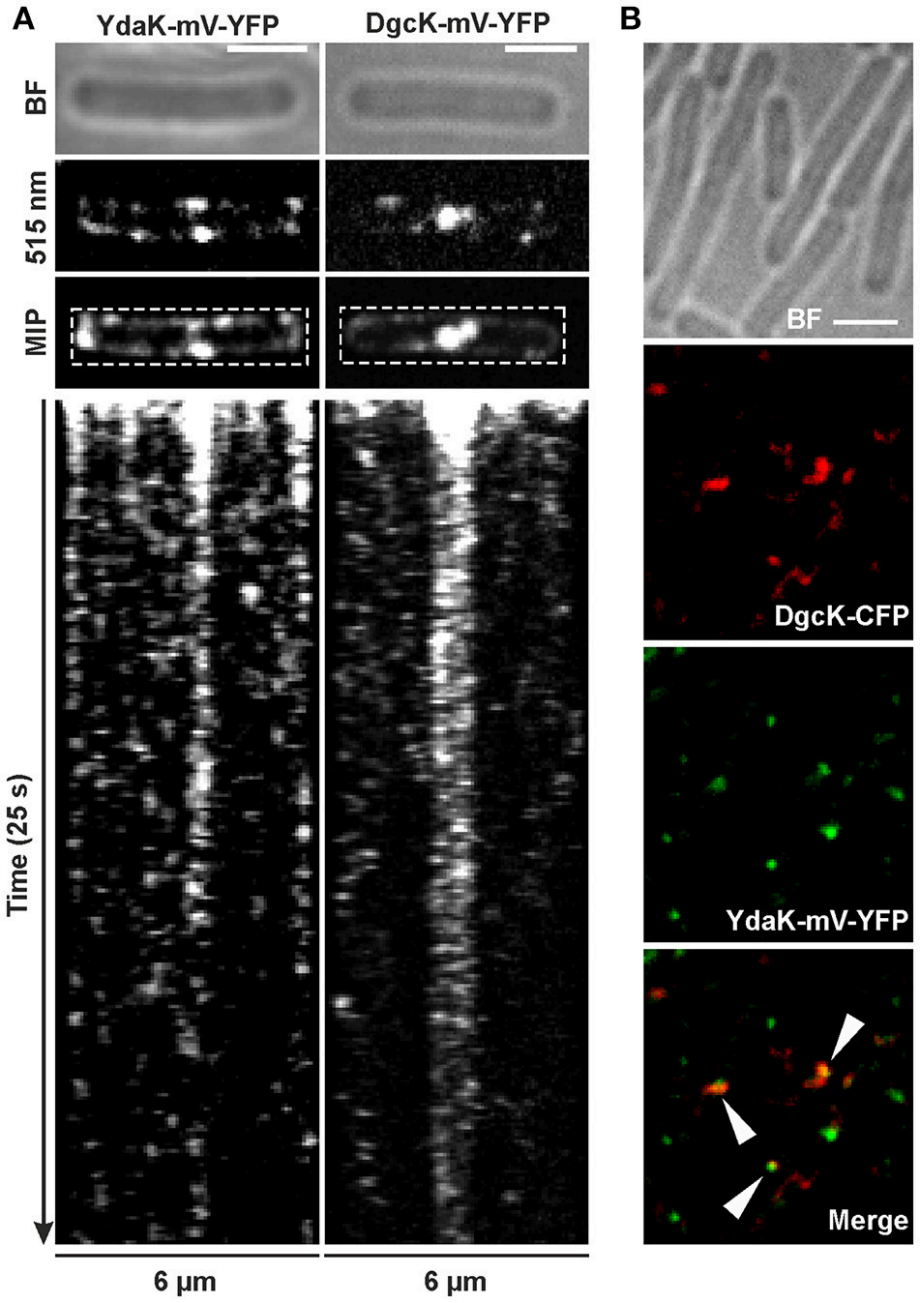
**Dynamics and simultaneous localization of YdaK and DgcK in ***B. subtilis*** NCIB3610. (A)** Representative time-lapse kymographs of YdaK-mV-YFP (left panel, strain NCIB3610-PB57; *amyE*::*P*_*xyl*_-*ydaK*-*mV-yfp*) and DgcK-mV-YFP (right panel, strain NCIB3610-PB90; *amyE*::*P*_*xyl*_-*dgcK*-*mV-yfp*) 45 min after induction with 0.1% xylose (v/v). BF: bright field (first row); snapshots (second row) and maximum intensity projection (MIP, third row) from time-lapse microscopy; fourth row: kymographs of fluorescence intensities along the rectangular selection depicted in the third row. Images were taken every 0.1 s upon continous illumination with 515 nm. **(B)** Co-localization of DgcK-CFP (445 nm, false colored red) originated from the ectopic *amyE* locus and YdaK-mV-YFP (515 nm, false-colored green) produced from the original locus, triangles indicate co-localization events (strain NCIB3610-PB37-PB10; *amyE*::*P*_*xyl*_-*dgcK*-*cfp, P*_*ydaK*_-*ydaK*-*mV-yfp*). Scale bars: 2 μm.

Similarly to our observation of YdaK-mV-YFP clustering (Figure 3Aiii), DgcK-mV-YFP fusion proteins also formed assemblies that retained a preference of localization to the cell poles and septa in NCIB3610 (Figure 3Ciii). This observation implies that DgcK and YdaK might be in spatial proximity at these cellular positions and might establish a spatially linked signal-effector cluster at the membrane. In addition to the comparable localization patterns of YdaK-mV-YFP and DgcK-mV-YFP (Figure 3), both fusion proteins exhibited a similar movement behavior when overproduced. We performed time-lapse experiments with both fusion proteins produced from the *amyE* locus upon xylose addition in exponentially growing *B. subtilis* NCIB3610 wild type cells. Upon continuous illumination (515 nm) with 100 ms intervals, foci detected at the septa were predominantly static, displaying negligible movement within a time span of several 100 ms (Figure 4A).

Polar foci were especially observed in case of YdaK-mV-YFP fusions and dynamic movement of both protein foci with lower “resting times” at the lateral cell periphery (Movies S1, S2). Although we observed events of co-localization in only 30% of total signals counted between DgcK-CFP (*amyE* locus) and YdaK-m-YFP (Figure 4B, strain NCIB3610-PB37-PB10, *amyE*::*P*_*xyl*_-*dgcK*-*cfp, P*_*ydaK*_-*ydaK*-*mV-yfp*), our data strongly suggest that c-di-GMP signaling and YdaK activation by DgcK occurs at the cell membrane and employs close spatial proximity of the players involved.

**Figure 5:**
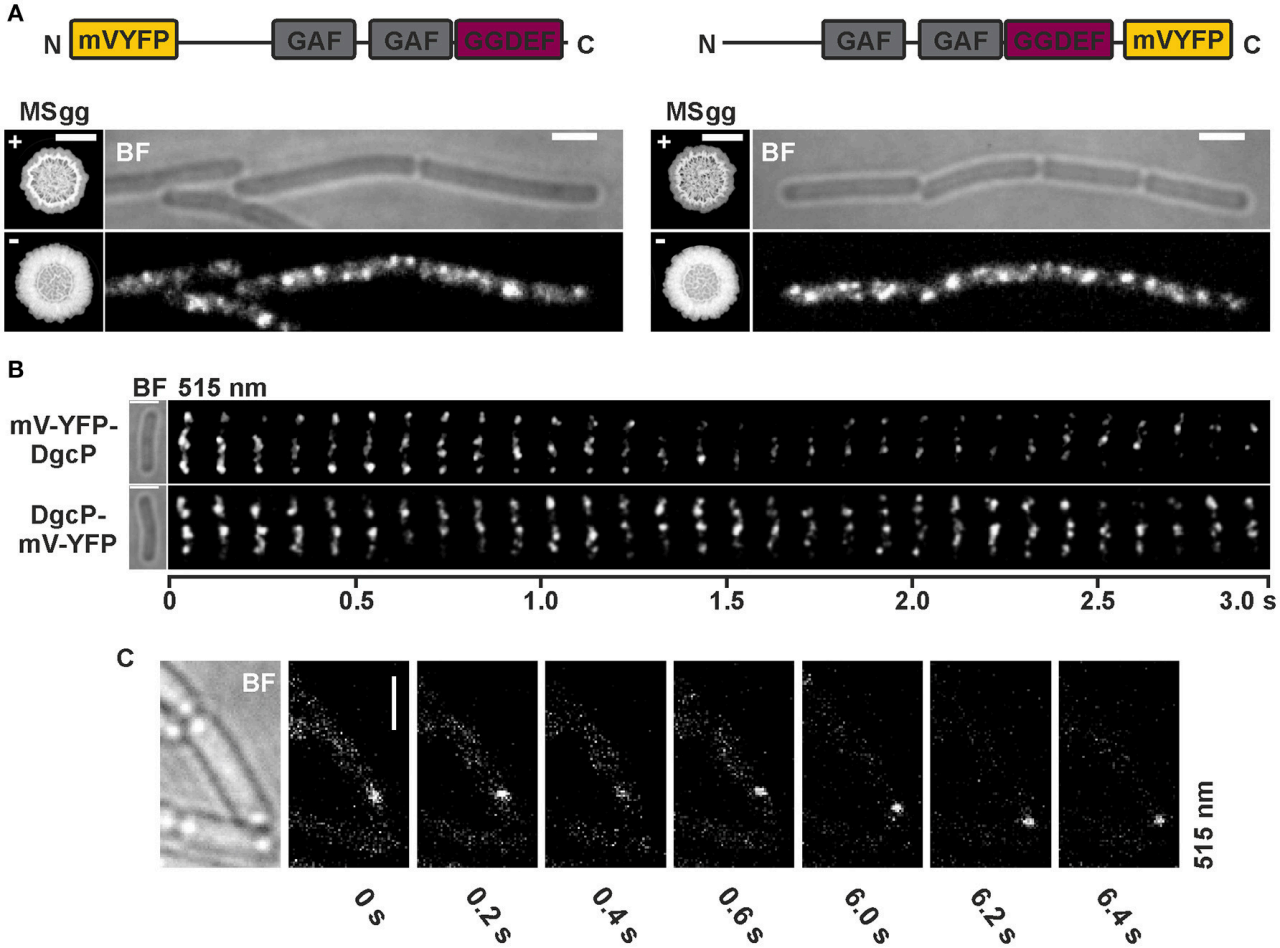
**Subcellular localization and dynamics of DgcP in ***B. subtilis*** NCIB3610. (A)** Epifluorescence of cells overexpressing *mV*-*yfp*-*dgcP* (left panel, strain NCIB3610-PB85; *amyE*::*P*_*xyl*_-*mV*-*yfp*-*dgcP*) and *dgcP*-*mV*-*yfp* (right panel, strain NCIB3610-PB86; *amyE*::*P*_*xyl*_-*dgcP*-*mV*-*yfp*) 45 min after induction with 0.1% (v/v) xylose (Scale bars: 2 μm). The “MSgg panel” depicts the functionality assay for the corresponding fusion protein/strain in the presence and absence of 0.1% (v/v) xylose (left: DS1809-PB55-PB85, Δ*dgcK*, -*P*, -*W, P*_*xyl*_-*ydaK*-*N, amyE*::*P*_*xyl*_-*mV*-*yfp*-*dgcP*; right: DS1809-PB55-PB86, Δ*dgcK*, -*P*, -*W, P*_*xyl*_-*ydaK*-*N, amyE*::*P*_*xyl*_-*dgcP*-*mV*-*yfp*). Scale bar: 5 mm. Color code for schematic representation of corresponding fusion protein (domain organization predicted by SMART): gray: GAF (domain found in cGMP-specific phosphodiesterases, adenylyl and guanylyl cyclases and phytochromes which often serves as a cyclic nucleotide binding domain), purple: GGDEF domain (active site motif GGEEL), yellow: N- and C-terminal mV-YFP respectively. **(B)** Time-lapse fluorescence microscopy of DgcP fusions produced. Images were captured every 100 ms under continious illumination (515 nm). Bars: 2 μm. **(C)** Time-lapse microscopy of DgcP-mV-YFP produced from the original locus (strain NCIB3610-PB08). Images were captured at the time points (seconds) indicated next to the panels at time intervals of 200 ms. Bars: 2 μm.

In addition to the localization of YdaK and DgcK, we also monitored mV-YFP labeled DgcP fusions in *B. subtilis* NCIB3610 (Figure 5, Figure S4). DgcP is a c-di-GMP synthesizing protein containing N-terminal GAF domains (putative sensor domain) and a C-terminal GGDEF domain, hinting that it is most likely a soluble protein in contrast to YdaK, DgcK, and DgcW. To test for the functionality of N- and C-terminal fusions, we applied the same complementation assay as described for DgcK (compare to Figure 3). Overexpression of *mV*-*yfp*-*dgcP* and of *dgcP*-*mV*-*yfp* respectively, in a DGC triple mutant inducing YdaK-N (strains: DS1809-PB55-PB85, Δ*dgcK*, -*P*, -*W, P*_*xyl*_-*ydaK*-*N, amyE*::*P*_*xyl*_-*mV*-*yfp*-*dgcP*; DS1809-PB55-PB86, Δ*dgcK*, -*P*, -*W, P*_*xyl*_-*ydaK*-*N, amyE*::*P*_*xyl*_-*dgcP*-*mV*-*yfp*) caused altered BF colony morphologies (Figures 5A,B, “MSgg panel”) in the same manner as seen for overexpression of *dgcK*-*mV*-*yfp* (Figure 3Bii).

Interestingly, stable mV-YFP-DgcP (Figure 5A, left panel, Figure S4) and DgcP-mV-YFP (Figure 5A, right panel, Figure S4) both assembled to subcellular clusters at the periphery of exponentially growing cells (produced from the ectopic *amyE* locus, strains NCIB3610-PB85, *amyE*::*P*_*xyl*_-*mV*-*yfp*-*dgcP*; NCIB3610-PB86, *amyE*::*P*_*xyl*_-*dgcP*-*mV*-*yfp*). Furthermore, both fusion proteins moved dynamically through the cell, but frequently arrested at the cell membrane for several 100 ms intervals (Figure 5B, Movie S3, Movie S4). A similar behavior was observed for DgcP-mV-YFP whose synthesis was initiated by its native promoter at its original locus resulting in low amounts of fusion proteins (Figure 5C, Figure S4). Thus, we conclude that DgcP would be able to deliver c-di-GMP to YdaK potentially through spatial proximity at the cell membrane. However, it is equally possible that YdaK is activated simply by elevated cytosolic c-di-GMP levels following the overproduction of DgcP (see deltaEAL-DgcW).

## Discussion

For Gram-positive bacteria, where the physiological role of c-di-GMP is not as well-characterized as in Gram-negative bacteria, c-di-GMP was demonstrated to influence, for example, swarming motility in the model organism *B*. *subtilis*, while it was not reported to have an effect on BF formation in this organism (Gao et al., 2013). Our study reveals that EPS production and therefore also potentially BF formation, are regulated by c-di-GMP, most likely post-translationally, via the effector protein YdaK (degenerated GGDEF domain protein), encoded within the putative EPS-synthesis operon *ydaJ*-*N* (Nicolas et al., 2012; Bedrunka and Graumann, 2017). The function of the unknown EPS in terms of BF formation, however, requires further examinations. Under our experimental conditions, YdaK is directed in its activity via DGC DgcK. We found that the presence of *dgc* genes and particularly the presence of *dgcK* and furthermore an intact conserved I-site motif (RxxD) of YdaK are indispensable for the production of an unknown EPS synthesized by YdaK-N on BF promoting medium, thereby revealing a new function for one of the three known DGC enzymes in *Bacillus subtilis*.

DgcK was first mentioned in the course of comparative genomic analysis revealing novel families of putative membrane-associated receptors (Anantharaman and Aravind, 2003). In this context, DgcK (formerly YhcK) and LytS from *B. subtilis* were selected to be the eponyms for the 5TMR –LYT family (for 5 transmembrane receptors of the LytS-YhcK type, PF07694) sharing a conserved membrane- spanning domain encompassing 5 TM helices harboring distinctive sequence features (ligand binding). Its mode of activation remains to be clarified. Interestingly, orthologs of DgcK [DgcA (Lmo1911) and DgcB (Lmo1912)] have been demonstrated to control ManNAc-Gal EPS synthesis (a β-1,4-linked N-acetylmannosamine chain decorated with α-1,6-linked galactose) via a degenerated GGDEF domain protein (PssE, encoded in the *pssABCDE* operon) in *Listeria monocytogenes* suggesting that this signaling cascade might be conserved in Gram-positive species (Chen et al., 2014; Koseoglu et al., 2015). However, members of the *B*. *cereus* group contain the ortholog gene *cdgA* (Fagerlund et al., 2016) but no operon similar to *ydaJ*-*M* of *B. subtilis*. Considering this, our study cannot exclude the possibility that DgcK affects other c-di-GMP signaling pathways even in *B. subtilis* via different effector proteins. The c-di-GMP receptor DgrA (PilZ domain protein) negatively influences swarming motility upon elevated intracellular c-di-GMP levels (Chen et al., 2012; Gao et al., 2013). However, the source of c-di-GMP “feeding” this receptor still needs to be elucidated. One could speculate that upon detection of an unknown signal (activation via 5TMR-LYT) DgcK activates both, DgrA and YdaK and thus swarming motility and EPS production respectively can be inversely regulated in order to mediate motile-to-sessile transition, the initial step of BF formation (Belas, 2014).

The direct regulation via DgcK notwithstanding, the activation of *B. subtilis* YdaK can also be accomplished by an overproduction of DgcP, which suggests that either DgcP has the potential to weakly activate the putative EPS machinery under normal conditions, or that globally elevated c-di-GMP concentrations due to the overproduction of the corresponding DGC are responsible for the activation of the machinery, i.e., that c-di-GMP signaling can be overcome by enhanced levels of a non-cognate DGC.

Moreover, overproduction of a truncated version of the third DGC, DgcW (a TM GGDEF-EAL tandem protein), lacking its C-terminal EAL domain also leads to an activation of YdaK. The construct mediated a transient cessation of swarming motility, when overexpressed in a GGDEF quadruple mutant, whereas overproduction of full length DgcW did not alter motility behavior via the PilZ domain protein DgrA (Gao et al., 2013). Thus, we suggest that an elimination of the EAL domain leads to elevated c-di-GMP concentrations in comparison to the WT protein and consequently activation of the *yda* operon can occur. On the other hand, we can exclude that DgcW influences the putative EPS machinery under physiological conditions because an overexpression of full length DgcW could not restore EPS production. Intriguingly, we found a new c-di-GMP-associated phenotype concerning biofilm formation during overexpression of truncated DgcW, in that we observed a profound effect on BF formation, suggesting that unbalanced production of c-di-GMP through DgcW interferes with biofilm maturation. The GGDEF domain of DgcW harbors a degenerated I-site motif (instead of RxxD, PxxG). Therefore, we hypothesize that the BF defect is a result of elevated c-di-GMP concentrations and thus a secondary effect, as DgcW-ΔEAL may not be subjected allosteric product inhibition in contrast to DgcK/P, or an elimination of the EAL domain results in an “exposure” of interaction sides for potential interaction partners/receptors of the GGDEF domain, thereby providing sufficient c-di-GMP concentrations that are limited in the WT protein by the adjacent EAL domain. It will be interesting to examine the potential involvement of the three proposed c-di-GMP receptors (DgrA, YdaK, YkuI) in the process of BF inhibition upon overproduction of DgcW-ΔEAL. Noteworthy, *dgcW* is assigned to the SigD regulon and co-expressed with various chemotaxis proteins (Nicolas et al., 2012; SubtiWiki) suggesting that DgcW might be linked to chemotaxis regulation potentially even through the activity of a yet unidentified c-di-GMP receptor in *B. subtilis*.

Besides the investigation of functions of DGCs in *B. subtilis*, we wanted to expand our knowledge on the dynamic behavior of GGDEF proteins in living cells. The presence of numerous DGC, PDE, and c-di-GMP receptor-encoding genes in various bacterial genomes raises questions regarding the mechanisms that establish specificity within these apparent diverse regulatory circuits (Hengge, 2009). One hypothesis proposes spatial proximity of c-di-GMP metabolizing proteins, effectors and targets, producing small localized specific concentrations as suggested in several studies (Merritt et al., 2010; Dahlstrom et al., 2015). We provide evidence that in *B. subtilis*, such a module shows subcellular clustering within the cell membrane. YdaK and DgcK co-localized to the same subcellular positions in the cell membrane establishing a potential c-di-GMP source-target network, which possibly ensures discrete c-di-GMP pools that are not utilized by other working modules, as already suggested for different other bacterial organisms (Guvener and Harwood, 2007; Ryan et al., 2010; Tan et al., 2014). Furthermore, both fusion proteins localized preferentially to the septa of exponentially growing cells where they exhibited high fluorescence intensities, indicating that both fusion might form higher oligomeric structures.

However, activation of EPS production can be also accomplished by potential non-cognate DGCs. Thus, it remains unclear whether c-di-GMP routes in *B. subtilis* do depend on central c-di-GMP hubs or whether they can be locally administrated (Valentini et al., 2016). For DgcP, we found diffusive movement within the cytosol, which contrasts the membrane-integral localization of DgcK, but interestingly, DgcP also arrested at many sites along the cell membrane, indicating that it may interact with a membrane-bound receptor. Enhanced levels of DgcP may thereby provide c-di-GMP directly to YdaK, but it is also possible that elevated cellular c-di-GMP levels activate YdaK in a non-specific manner. This idea is supported by the finding that overproduced DgcW lacking the EAL domain can also lead to an activation of the *yda* operon. Therefore, both local and global c-di-GMP pools appear to play important roles in signaling pathways in *B. subtilis*.

It will be interesting to further investigate *in vivo* dynamics of DGCs, or their receptors and of their regulated proteins, in order to obtain a more detailed view of the molecular mechanism operating based on local and/ or global c-di-GMP signaling.

## Materials and methods

### General methods and bacterial growth conditions

DNA manipulation and *Escherichia coli* DH5α transformations were carried out using standard techniques (Mamiatis et al., 1985; Gibson et al., 2009). *E. coli* strains were routinely cultivated at 37°C in Lysogeny Broth (LB) medium supplemented with 100 μg ml^−1^ ampicillin. Lists of utilized plasmids and oligonucleotides are provided in Tables S2, S3 respectively. All constructs were verified by DNA sequencing.

*Bacillus subtilis* strains used in this study derived from the non-domesticated strain NCIB3610 (BGSC) or its transformable derivative DK1042 (Gift from D. Kearns). For transformation of both classes of derivatives, *B. subtilis* overnight cultures were grown in liquid LB at 30°C and were diluted to OD_600_ 0.08 in 10 ml of a modified competence medium (Zafra et al., 2012). Inoculated cells were further incubated at 37°C and 200 rpm. Upon entry into stationary phase (OD_600_ 1.4–1.6), 300–500 ng of purified genomic DNA were added to 1 ml culture of NCIB3610 derivatives and 0.5-1 μg of plasmid DNA to 1 ml culture of DK1042 derivatives respectively. Cells were further incubated at 37°C and 200 rpm for 2 h followed by selection on solid medium with the appropriate antibiotic. Final antibiotic concentrations were: 5 μg ml^−1^ chloramphenicol, 50–100 μg ml^−1^ spectinomycin and 10 μg ml^−1^ tetracycline. Table S1 provides a detailed description of strains and whether they were generated by plasmid- or by chromosomal DNA-transformation.

For routine growth, *B. subtilis* cells were streaked from frozen stocks onto LB agar plates and incubated overnight at 37°C. Overnight cultures were grown at 30°C and 200 rpm in LB under antibiotic selective pressure and at 37°C previous to phenotypical tests and fluorescence microscopy. Prior to microscopy cells were washed twice in S7_50_ minimal medium containing 1.0% (v/v) fructose, 0.1% (v/v) glutamate, 0.004% (v/v) casamino acids (Jaacks et al., 1989). For induction of the xylose promoter, xylose was added up to 0.1% (v/v). For induction of the hyperspank promoter, the culture medium was supplemented with 1 mM isopropyl-β-D-thiogalactopyranoside (IPTG).

### Strain construction

#### *yda* expression constructs

To generate strains overexpressing the transcriptional unit *ydaJ*-*N* or truncated variants of it (*ydaK*-*N, ydaL*-*N*), we used three overexpression constructs: pSG1164-PB53, -PB55, -PB56 (Bedrunka and Graumann, 2017). Recombinant plasmids were introduced into *B. subtilis* NCIB3610 (DK1042) by genetic transformation (Zafra et al., 2012). Correct single-crossover plasmid integration into the host genome was verified by PCR using a specific xylose promoter binding primer (PG5050f) and a primer complementary to the distal end of the operon (PB19r) as already described in Bedrunka and Graumann (2017). For integration of the corresponding constructs into the genomes of NCIB3610 derived *dgc* mutants, which were kindly provided by Charles Dann III and Daniel Kearns (Indiana), first chromosomal DNA from the corresponding DK1042 strains was isolated and subsequently 300–500 ng of purified DNA was transformed into the corresponding *dgc* mutant backgrounds. The maintenance of gene deletions was verified via PCR using the oligonucleotides (2928–3040) listed in Table S3.

#### Complementation strains

Overexpression constructs of *ydaK, dgcK, dgcP, dgcW* and of *dgcW*Δ*eal* under the control of a hyperspank promotor at the *amyE* site respectively (pXG003, pXG004, pXG002, pXG001, and pXG086) were kindly provided by the Labs of Charles Dann III and Daniel Kearns (Indiana). The corresponding constructs were integrated first into the genomes of *B. subtilis* NCIB3610 (DK1042) by plasmid transformation. A double-crossover recombination of the DNA sequences at the *amyE* locus on the chromosomes was confirmed by screening loss of starch degradation. To overexpress the corresponding genes in the NCIB3610 derived *dgc* mutant strains which also overexpress the *ydaK*-*N* operon (strain DS1809-PB55), chromosomal DNA was isolated from DK1042 derivatives and further transformed into the corresponding mutant strains (300–500 ng). Mutations R202A and D204A in YdaK were created using a PCR-based site-directed mutagenesis kit (Q5 Site-Directed Mutagenesis Kit, NEB). Mutagenesis was performed on plasmid pXG003 carrying the *ydaK* gene using primer pairs PB80f/PB80r for R202A and PB81f/PB81r for D204A, respectively. Mutant alleles were fully sequenced to verify mutations.

#### Xylose-inducible translational fluorescent fusions (at *amyE*)

To generate inducible translational C-terminal fusions of YdaK, YdaKΔ4TMH, DgcK, DgcP and DgcW to mVenus-YFP (Venus YFP with monomerizing A206K mutation) or CFP, the corresponding coding sequences missing the stop codon, were amplified by PCR using NCIB3610 chromosomal DNA as a template and primer pairs PB57f/PB57r for *ydaK*-*mV*-*yfp*, PB100f/PB57r for *ydaK*Δ4*tmh*-*mV*-*yfp*, PB90f/PB90r for *dgcK*-*mV*-*yfp* and PB21f/PB37r *dgcK*-*cfp*, PB86f/PB86r for *dgcP*-*mV*-*yfp* and PB88f/PB88r for *dgcW*-*mV*-*yfp*, respectively (Table S3). The resulting fragments were digested and ligated into the corresponding sites of pSG1193-NLMV containing a spectinomycin resistance cassette, a polylinker downstream of the xylose promoter, and the gene encoding mV-YFP between the two arms of the *amyE* gene. For C-terminal CFP fusions, *dgcK* was cloned into pSG1192 (Lewis and Marston, 1999). The resulting recombinant plasmids are listed in Table S2. Constructs for N-terminal YFP fusions of DgcK, DgcP, and DgcW were generated in a similar manner using oligonucleotides PB21f/PB79r for *dgcK*, PB20f/PB85r for *dgcP* and PB87f/PB87r for *dgcW* and plasmid pSG1729-MVYFP. Recombinant plasmids were introduced into the *B. subtilis* strain DK1042 by genetic transformation. A double-crossover recombination of the DNA sequences at the *amyE* locus on the chromosomes was confirmed by screening loss of starch degradation and stability of fusion proteins was verified by immune-detection using anti-GFP serum (see below). To overexpress fusion genes in NCIB3610 derived *dgc* mutant strains which also overexpress the truncated *ydaKLMN* operon (strain DS1809-PB55) and to test for their functionality, chromosomal DNA was isolated from DK1042 derivatives and further transformed into the corresponding mutants (300–500 ng).

#### Fusion proteins encoded at the original locus

To obtain *mV*-*yfp* gene fusions of *dgcK, ydaK*, and *dgcP*, which are encoded at the endogenous locus and expression is driven by the respective original promotor, a minimum of 500 bp of the 3′ region of the genes was amplified first by PCR using the oligonucleotides, PB01/f/PB01r, PB10f/PB10r, and PB08f/PB08r respectively. The resulting fragments were cloned into plasmid pSG1164-NLMV using overlapping sequences [isothermal “Gibson” assembly (ITA), Gibson et al., 2009]. Competent cells of DK1042 were transformed with the plasmids pSG1164-NLMV-PB01, pSG1164-NLMV-PB10, and pSG1164-NLMV-PB08 generating the strains listed in Table S1. For Co-localization studies of YdaK-mV-YFP and DgcK-CFP, plasmid pSG1164-NLMV-PB10 was transformed into NCIB3610-PB37 (*amyE*::*P*_*xyl*_-*dgcK*-*cfp*) resulting in strain NCIB3610-PB37-PB10.

### Biofilm formation assay

Undomesticated *B. subtilis* NCIB3610 strains were cultured in LB containing appropriate antibiotics at 30°C for 14 h. Daily cultures were grown in LB at 37°C to an OD_600_ of 1.0 without antibiotics. For biofilm growth, bacteria from a liquid LB culture were collected and transferred to liquid MSgg medium [5 mM potassium phosphate (pH 7.0), 100 mM 3-(*N*-morpholino) propane-sulfonic acid (pH 7.0), 2 mM MgCl_2_, 700 μM CaCl_2_, 50 μM MnCl_2_, 100 μM FeCl_3_, 1 μM ZnCl_2_, 2 μM thiamine, 0.5% glycerol, 0.5% glutamate]. Cells were incubated for additional 30 min at 37°C and 200 rpm before inoculation (2 μl) on MSgg plates (MSgg medium fortified with 1.5% Bacto agar, 6- well plates, dried overnight) supplemented with Congo Red (40 μg ml^−1^) and Coomassie Brilliant Blue (20 μg ml^−1^) and with or without 0.1% (v/v) xylose and or 1 mM IPTG (Branda et al., 2001; Asally et al., 2012). Plates were sealed and incubated up to 72 h at 25°C. Colony morphology was documented over time using the ChemiDocTM MP System (BIO-RAD). For each strain, we analyzed 3 biological replicates in at least two independent experiments.

### Fluorescence microscopy

Cells were grown in LB rich medium under selective pressure to the exponential growth phase at 37°C. D-xylose was added in different concentrations [0.001, 0.01, and 0.1% (v/v)] to the growth media to induce expression of genes downstream of the encoded fusion protein at original locus or the encoded fusion protein itself at the *amyE* locus for 45 min at 37°C. For microscopy, 2 μl of washed cells were spotted on a coverslip and immobilized by a thin agarose pad [1% (w/v) agarose in S7_50_ minimal medium]. Fluorescence microscopy was performed using a Zeiss Axio Observer A1 equipped with a 100 × TIRF objective (numerical aperture NA of 1.45) using the setup from Visitron Systems (Munich, Germany). YFP fluorophores were excited by exposure to a 515 nm laser beam and CFP fluorophores to 445 nm. Images were acquired with an Evolve EM-CCD camera (Photometrix) and were processed with ImageJ (National Institutes of Health, Bethesda, MD).

### Immunoblotting

To validate expression levels of fusion genes and stability of fusion proteins respectively, cells were grown in LB medium at 37°C until exponential phase and gene expression was artificially induced with different xylose concentrations in case of *amyE* encoded gene fusions. 45 min after induction and incubation at 37°C and 200 rpm, equal amounts of cells were resuspended in lysis buffer (50 mM EDTA, 100 mM NaCl, 2.5 mg ml^−1^ lysozyme, 0.1 mg ml^−1^ RNase, 0.01 mg ml^−1^ DNase, pH 7.5) and incubated for 20 min at 37°C. SDS sample buffer (final concentration, 1 X) was added to the cell lysate and boiled at 95°C for 10 min, except for lysates which derived from strain NCIB3610-PB90 (*amyE*::*P*_*xyl*_-*dgcK*-*mV*-*yfp*). These samples were incubated at RT for 1 h prior to SDS-PAGE on 4–20% Tris/ Glycin gradient gels which have been also used for separation of YdaK fusion proteins. DgcP fusion proteins were separated via SDS-PAGE on 12% gels. The proteins were transferred to a nitrocellulose membrane, applying the semidry Western blotting method for 1 h and 45 mA and YFP-fused proteins were visualized by a primary polyclonal α-GFP antiserum (dilution, 1:500) and a secondary goat α-rabbit antiserum coupled to a horseradish peroxidase.

## Author contributions

PB has performed and analyzed all experiments, has conceived of the experiments and has written the manuscript. PG has conceived of the experiments, and has written the manuscript.

### Conflict of interest statement

The authors declare that the research was conducted in the absence of any commercial or financial relationships that could be construed as a potential conflict of interest.

## References

[B1] Anantharaman V. Aravind L. (2003). Application of comparative genomics in the identification and analysis of novel families of membrane-associated receptors in bacteria. BMC Genomics 4:34. 10.1186/1471-2164-4-34 12914674 PMC212514

[B2] Asally M. Kittisopikul M. Rue P. Du Y. Hu Z. Cagatay T.. (2012). Localized cell death focuses mechanical forces during 3D patterning in a biofilm. Proc. Natl. Acad. Sci. U.S.A. 109, 18891–18896. 10.1073/pnas.1212429109 23012477 PMC3503208

[B3] Bedrunka P. Graumann P. L. (2017). Subcellular clustering of a putative c-di-GMP-dependent exopolysaccharide machinery affecting macro colony architecture in *Bacillus subtilis*. Environ. Microbiol. Rep. [Epub ahead of print]. 10.1111/1758-2229.12496 27897378

[B4] Belas R. (2014). Biofilms, flagella, and mechanosensing of surfaces by bacteria. Trends Microbiol. 22, 517–527. 10.1016/j.tim.2014.05.002 24894628

[B5] Branda S. S. Gonzalez-Pastor J. E. Ben-Yehuda S. Losick R. Kolter R. (2001). Fruiting body formation by *Bacillus subtilis*. Proc. Natl. Acad. Sci. U.S.A. 98, 11621–11626. 10.1073/pnas.191384198 11572999 PMC58779

[B6] Chen L. H. Koseoglu V. K. Guvener Z. T. Myers-Morales T. Reed J. M. D'Orazio S. E.. (2014). Cyclic di-GMP-dependent signaling pathways in the pathogenic Firmicute *Listeria monocytogenes*. PLoS Pathog. 10:e1004301. 10.1371/journal.ppat.1004301 25101646 PMC4125290

[B7] Chen Y. Chai Y. Guo J. H. Losick R. (2012). Evidence for cyclic Di-GMP-mediated signaling in *Bacillus subtilis*. J. Bacteriol. 194, 5080–5090. 10.1128/JB.01092-12 22821967 PMC3430322

[B8] Christen B. Christen M. Paul R. Schmid F. Folcher M. Jenoe P.. (2006). Allosteric control of cyclic di-GMP signaling. J. Biol. Chem. 281, 32015–32024. 10.1074/jbc.M603589200 16923812

[B9] Dahlstrom K. M. Giglio K. M. Collins A. J. Sondermann H. O'Toole G. A. (2015). Contribution of physical interactions to signaling specificity between a diguanylate cyclase and its effector. MBio 6, e01978–e01915. 10.1128/mBio.01978-15 26670387 PMC4676286

[B10] Dahlstrom K. M. Giglio K. M. Sondermann H. O'Toole G. A. (2016). The inhibitory site of a diguanylate cyclase is a necessary element for interaction and signaling with an effector protein. J. Bacteriol. 198, 1595–1603. 10.1128/JB.00090-16 27002135 PMC4959289

[B11] De N. Pirruccello M. Krasteva P. V. Bae N. Raghavan R. V. Sondermann H. (2008). Phosphorylation-independent regulation of the diguanylate cyclase WspR. PLoS Biol. 6:e67. 10.1371/journal.pbio.0060067 18366254 PMC2270323

[B12] Fagerlund A. Smith V. Rohr A. K. Lindback T. Parmer M. P. Andersson K. K.. (2016). Cyclic diguanylate regulation of *Bacillus cereus* group biofilm formation. Mol. Microbiol. 101, 471–494. 10.1111/mmi.13405 27116468

[B13] Gao X. Mukherjee S. Matthews P. M. Hammad L. A. Kearns D. B. Dann C. E. III. (2013). Functional characterization of core components of the *Bacillus subtilis* cyclic-di-GMP signaling pathway. J. Bacteriol. 195, 4782–4792. 10.1128/JB.00373-13 23893111 PMC3807487

[B14] Gibson D. G. Young L. Chuang R. Y. Venter J. C. Hutchison C. A. III. Smith H. O. (2009). Enzymatic assembly of DNA molecules up to several hundred kilobases. Nat. Methods 6, 343–345. 10.1038/nmeth.1318 19363495

[B15] Gomelsky M. (2011). cAMP, c-di-GMP, c-di-AMP and now cGMP: bacteria use them all! Mol. Microbiol. 79, 562–565. 10.1111/j.1365-2958.2010.07514.x 21255104 PMC3079424

[B16] Guvener Z. T. Harwood C. S. (2007). Subcellular location characteristics of the *Pseudomonas aeruginosa* GGDEF protein, WspR, indicate that it produces cyclic-di-GMP in response to growth on surfaces. Mol. Microbiol. 66, 1459–1473. 10.1111/j.1365-2958.2007.06008.x 18028314 PMC4105145

[B17] Hengge R. (2009). Principles of c-di-GMP signalling in bacteria. Nat. Rev. Microbiol. 7, 263–273. 10.1038/nrmicro2109 19287449

[B18] Hengge R. Grundling A. Jenal U. Ryan R. Yildiz F. (2016). Bacterial signal transduction by cyclic di-GMP and other nucleotide second messengers. J. Bacteriol. 198, 15–26. 10.1128/JB.00331-15 26055111 PMC4686208

[B19] Jaacks K. J. Healy J. Losick R. Grossman A. D. (1989). Identification and characterization of genes controlled by the sporulation-regulatory gene spo0H in *Bacillus subtilis*. J. Bacteriol. 171, 4121–4129. 2502532 10.1128/jb.171.8.4121-4129.1989 PMC210181

[B20] Jenal U. Reinders A. Lori C. (2017). Cyclic di-GMP: second messenger extraordinaire. Nat. Rev. Microbiol. 15, 271–284. 10.1038/nrmicro.2016.190 28163311

[B21] Kearns D. B. Chu F. Branda S. S. Kolter R. Losick R. (2005). A master regulator for biofilm formation by *Bacillus subtilis*. Mol. Microbiol. 55, 739–749. 10.1111/j.1365-2958.2004.04440.x 15661000

[B22] Koseoglu V. K. Heiss C. Azadi P. Topchiy E. Guvener Z. T. Lehmann T. E.. (2015). *Listeria monocytogenes* exopolysaccharide: origin, structure, biosynthetic machinery and c-di-GMP-dependent regulation. Mol. Microbiol. 96, 728–743. 10.1111/mmi.12966 25662512

[B23] Lewis P. J. Marston A. L. (1999). GFP vectors for controlled expression and dual labelling of protein fusions in *Bacillus subtilis*. Gene 227, 101–110. 9931458 10.1016/s0378-1119(98)00580-0

[B24] Liang Z. X. (2015). The expanding roles of c-di-GMP in the biosynthesis of exopolysaccharides and secondary metabolites. Nat. Prod. Rep. 32, 663–683. 10.1039/c4np00086b 25666534

[B25] Lindenberg S. Klauck G. Pesavento C. Klauck E. Hengge R. (2013). The EAL domain protein YciR acts as a trigger enzyme in a c-di-GMP signalling cascade in *E. coli* biofilm control. EMBO J. 32, 2001–2014. 10.1038/emboj.2013.120 23708798 PMC3715855

[B26] Mamiatis T. Fritsch E. F. Sambrook J. Engel J. (1985). Molecular cloning–A laboratory manual. New York: Cold Spring Harbor Laboratory. 1982, 545 S. Acta Biotechnol. 5, 104–104. 10.1002/abio.370050118

[B27] Merritt J. H. Ha D. G. Cowles K. N. Lu W. Morales D. K. Rabinowitz J.. (2010). Specific control of *Pseudomonas aeruginosa* surface-associated behaviors by two c-di-GMP diguanylate cyclases. MBio 1:e00183–10. 10.1128/mBio.00183-10 20978535 PMC2957078

[B28] Nicolas P. Mader U. Dervyn E. Rochat T. Leduc A. Pigeonneau N.. (2012). Condition-dependent transcriptome reveals high-level regulatory architecture in *Bacillus subtilis*. Science 335, 1103–1106. 10.1126/science.1206848 22383849

[B29] Plate L. Marletta M. A. (2012). Nitric oxide modulates bacterial biofilm formation through a multicomponent cyclic-di-GMP signaling network. Mol. Cell 46, 449–460. 10.1016/j.molcel.2012.03.023 22542454 PMC3361614

[B30] Romling U. Galperin M. Y. Gomelsky M. (2013). Cyclic di-GMP: the first 25 years of a universal bacterial second messenger. Microbiol. Mol. Biol. Rev. 77, 1–52. 10.1128/MMBR.00043-12 23471616 PMC3591986

[B31] Ryan R. P. McCarthy Y. Andrade M. Farah C. S. Armitage J. P. Dow J. M. (2010). Cell-cell signal-dependent dynamic interactions between HD-GYP and GGDEF domain proteins mediate virulence in *Xanthomonas campestris*. Proc. Natl. Acad. Sci. U.S.A. 107, 5989–5994. 10.1073/pnas.0912839107 20231439 PMC2851925

[B32] Ryan R. P. Tolker-Nielsen T. Dow J. M. (2012). When the PilZ don't work: effectors for cyclic di-GMP action in bacteria. Trends Microbiol. 20, 235–242. 10.1016/j.tim.2012.02.008 22444828

[B33] Schirmer T. Jenal U. (2009). Structural and mechanistic determinants of c-di-GMP signalling. Nat. Rev. Microbiol. 7, 724–735. 10.1038/nrmicro2203 19756011

[B34] Serra D. O. Richter A. M. Hengge R. (2013). Cellulose as an architectural element in spatially structured *Escherichia coli* biofilms. J. Bacteriol. 195, 5540–5554. 10.1128/Jb.00946-13 24097954 PMC3889604

[B35] Tan H. West J. A. Ramsay J. P. Monson R. E. Griffin J. L. Toth I. K.. (2014). Comprehensive overexpression analysis of cyclic-di-GMP signalling proteins in the phytopathogen *Pectobacterium atrosepticum* reveals diverse effects on motility and virulence phenotypes. Microbiology 160, 1427–1439. 10.1099/mic.0.076828-0 24760967

[B36] Valentini M. Laventie B. J. Moscoso J. Jenal U. Filloux A. (2016). The diguanylate cyclase HsbD intersects with the HptB regulatory cascade to control *Pseudomonas aeruginosa* biofilm and motility. PLoS Genet. 12:e1006354. 10.1371/journal.pgen.1006354 27792789 PMC5085249

[B37] Zafra O. Lamprecht-Grandio M. de Figueras C. G. Gonzalez-Pastor J. E. (2012). Extracellular DNA release by undomesticated *Bacillus subtilis* is regulated by early competence. PLoS ONE 7:e48716. 10.1371/journal.pone.0048716 23133654 PMC3487849

[B38] Zahringer F. Lacanna E. Jenal U. Schirmer T. Boehm A. (2013). Structure and signaling mechanism of a zinc-sensory diguanylate cyclase. Structure 21, 1149–1157. 10.1016/j.str.2013.04.026 23769666

[B39] Zhu B. Liu C. Liu S. Cong H. Chen Y. Gu L.. (2016). Membrane association of SadC enhances its diguanylate cyclase activity to control exopolysaccharides synthesis and biofilm formation in *Pseudomonas aeruginosa*. Environ. Microbiol. 18, 3440–3452. 10.1111/1462-2920.13263 26940526

