# Supplementary material for: New Functions and Subcellular Localization Patterns of c-di-GMP Components (GGDEF Domain Proteins) in B. subtilis
Source: Front Microbiol. 2017 May 9;8:794. doi: 10.3389/fmicb.2017.00794 (PMC5422440; doi:10.3389/fmicb.2017.00794)
Supplement: Supplementary file 5 [file DataSheet1.docx]

**Supplemental Material/ Information**

**New functions and subcellular localization of c-di-GMP components (GGDEF domain proteins) in *B*. *subtilis***

Patricia Bedrunka and Peter L. Graumann*

LOEWE SYNMIKRO, LOEWE Center for Synthetic Microbiology and Department of Chemistry, Philipps University Marburg, Hans-Meerwein Strasse, 35043 Marburg, Germany

**Movie S1:** Fluorescence microscopy of *B. subtilis* NCIB3610 producing YdaK-mV-YFP

**Movie S2:** Exponential *B. subtilis* NCIB3610 producing DgcK-mV-YFP

**Movie S3:** Exponential *B. subtilis* NCIB3610 expressing *mV*-*yfp*-*dgcP*

**Movie S4:** Exponential *B. subtilis* NCIB3610 expressing *dgcP*-*mV*-*yfp*

Time intervals: 100 ms upon continuous illumination with 515 nm

All movies are played at 15 fps (frames per second)


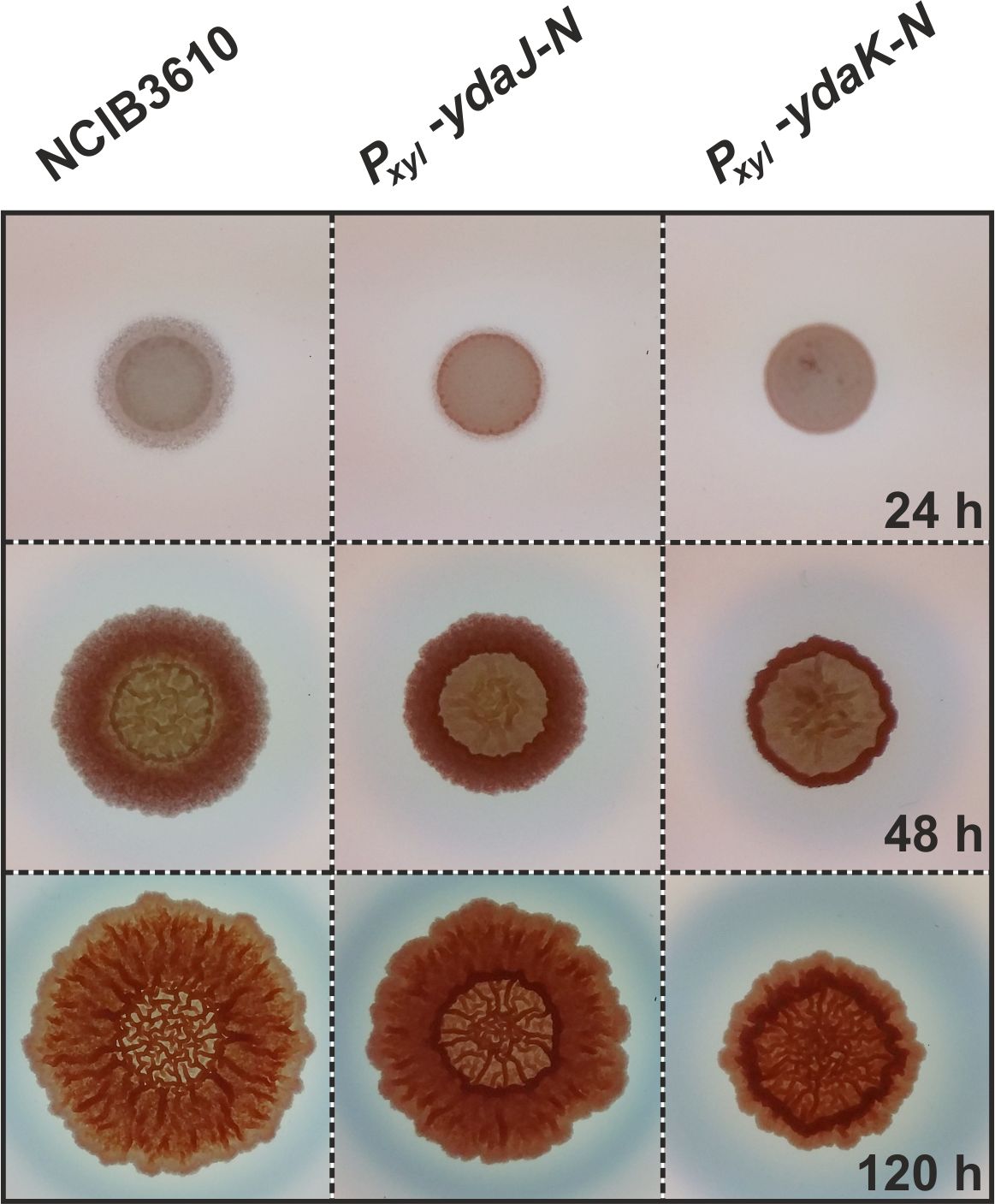


**Fig S1.**

**Overexpression of the *ydaJ*-*N*- and *ydaK*-*N*- operon variants results in increased Congo**

**Red staining and altered BF colony morphologies of *B*. *subtilis* NCIB3610**

Individual clones of the indicated strains (WT NCIB3610; NCIB3610-PB53: *P_xyl_*-*ydaJ*-*N*; NCIB3610-PB55: *P_xyl_*-*ydaK*-*N*) were grown in LB at 37 °C. An aliquot of mid-log cultures was spotted on MSgg agar plates supplemented with 0.1 % (v/v) xylose, CR 40 µg/ml, CB 20 µg/ml, following incubation at 28 °C. Imaging of colonies was carried out at the indicated time points using a light screen.


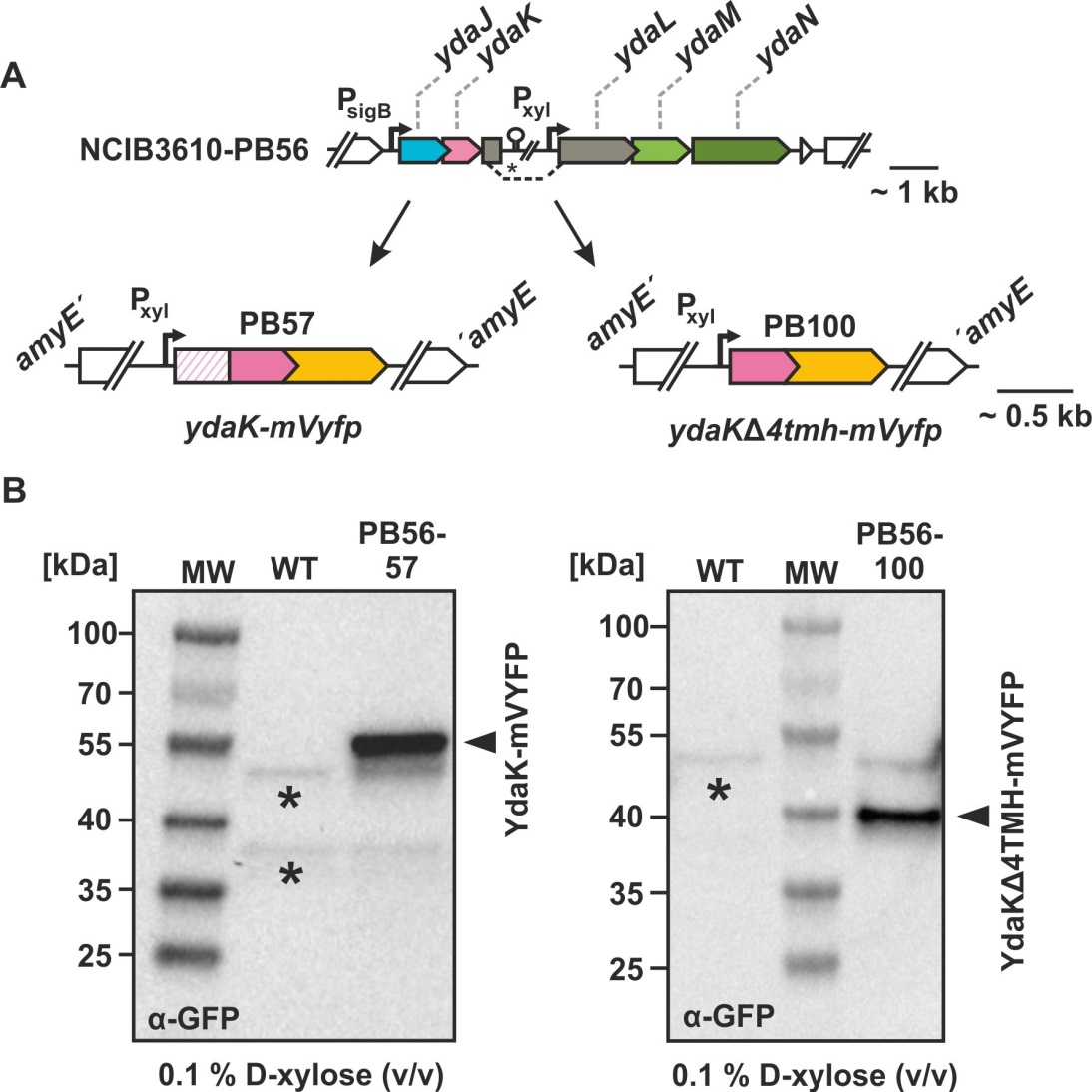


**Fig S2.**

**Verification of YdaK-fusion proteins by Western Blotting**

(**A**) Schematic representation of the ydaJ-N and amyE genomic locus in strains overexpressing ydaLMN/ ydaK-mV-yfp (left, strain: NCIB3610-PB56-PB57; *P_xyl_*-*ydaL*-*N*, *amyE*::*P_xyl_*-*ydaK*-*mV*-*yfp*) and ydaLMN/ ydaKΔ*4tmh*-mV-yfp (right, strain: NCIB3610-PB56-PB100, *P_xyl_*-*ydaL*-*N*, *amyE*::*P_xyl_*-*ydaK*Δ*4tmh*-*mV-yfp*) respectively. Dashed line represents the integrated plasmid pSG1164-PB56 into the genome of NCIB3610. Star indicates an insertional point mutation in the construct resulting in Δ*ydaJK*. (**B**) Immuno-detection of YdaK-mV-YFP (left) and of the truncation mutant YdaKΔ4TMH-mV-YFP (right) in total cell extracts of the indicated strains WT (wild type NCIB3610), NCIB3610-PB56-PB57 and NCIB-PB56-PB100 respectively, using anti-GFP antiserum. Gene expression was induced at OD_600_ 0.45 for 45 min at 37 °C with 0.1 % xylose (v/v). All lanes are normalized to optical cell density. Separation of proteins via SDS-PAGE was performed on 4-20 % polyacrylamide gels prior to transfer on a nitrocellulose membrane by the semidry Western blotting method. The asterisks in the wild type control indicate cross-reacting species present in all lanes. The calculated sizes of the two fusion proteins are 59 kDa, for YdaK-mV-YFP and 42 kDa for YdaKΔ4TMH-mV-YFP.

**
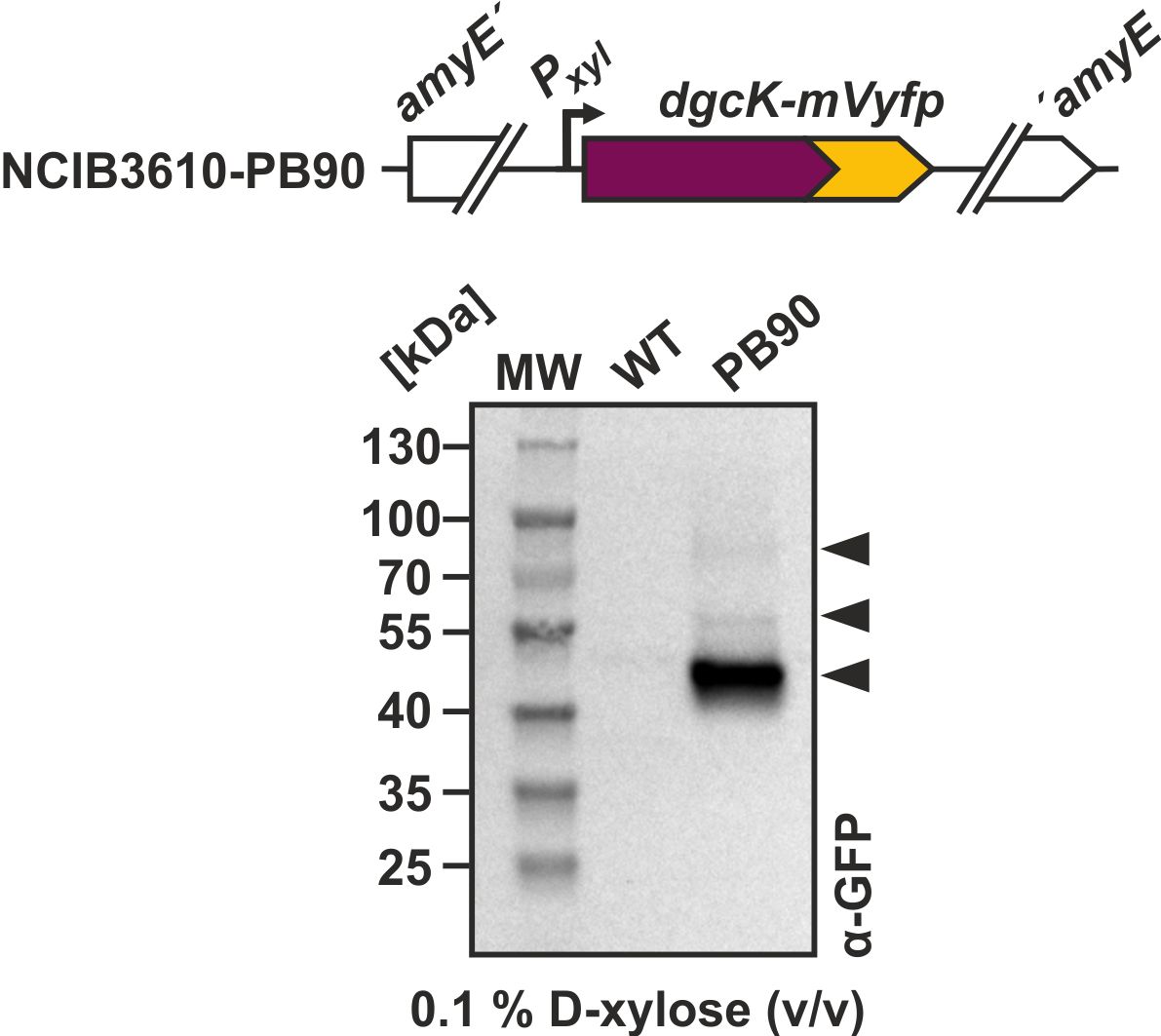
**

**Fig S3.**

**Immunodetection of DgcK-mV-YFP using α-GFP antiserum**

Scheme of a*myE* gene locus in strain NCIB3610-PB90 overexpressing *dgcK*-*mV*-*yfp* and detection of C-terminal mV-YFP fusions of DgcK. Expression was induced at OD_600_ 0.5 for 45 min at 37 °C. Lysed cells were incubated with SDS sample buffer for 45 min at RT. Separation of proteins from equal amounts of cells via SDS-PAGE was performed on 4-20 % polyacrylamide gels prior to Western blotting. Arrows indicate position of DgcK-mV-YFP fusions (estimated size 67 kDa). Note that DgcK-mV-YFP runs aberrantly during SDS-PAGE.


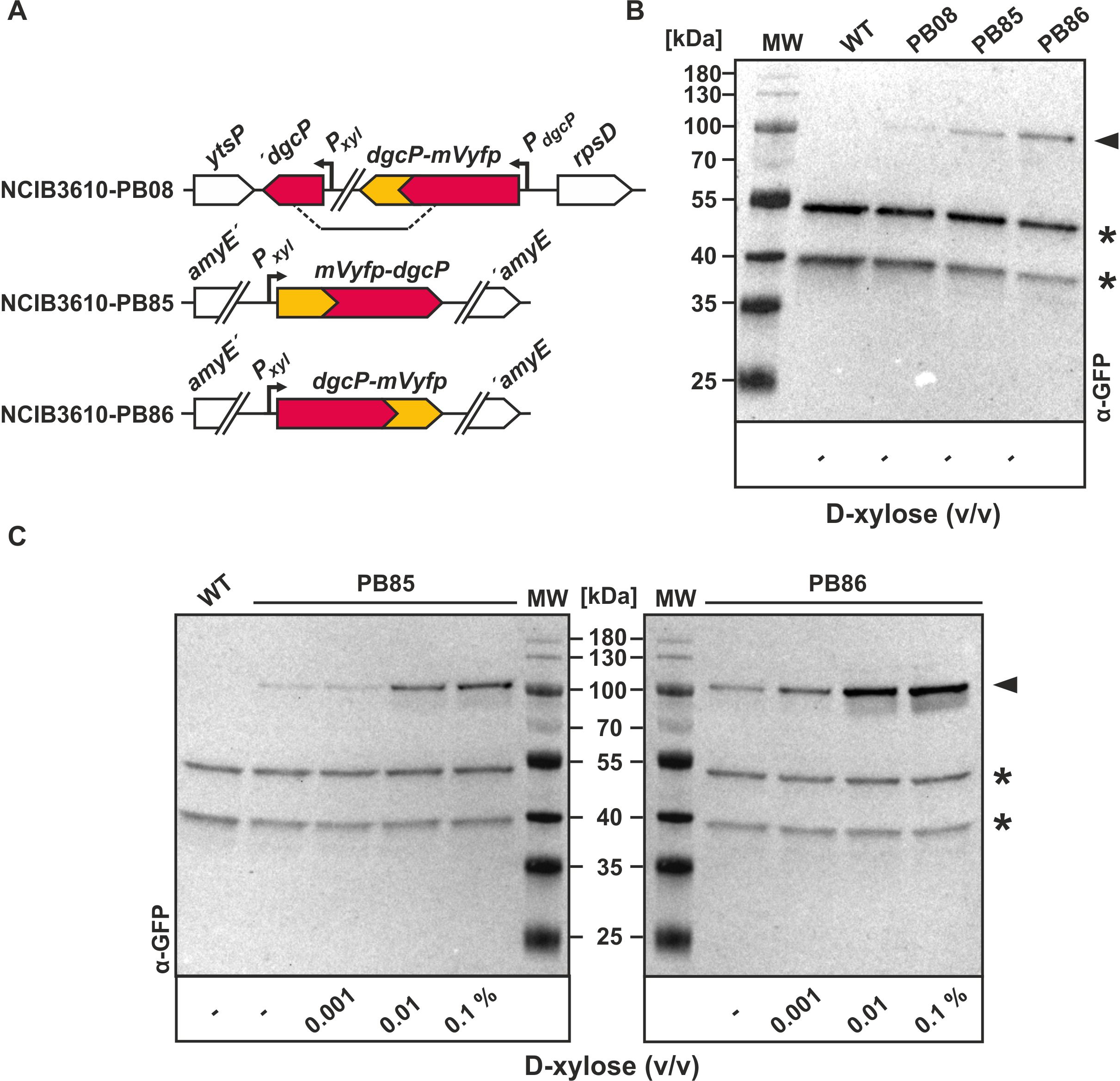


**Fig S4.**

**Analysis of DgcP fusion proteins via immunodetection with α-GFP antiserum**

(**A**) Schematic representation of *mV*-*yfp* gene fusions for strains NCIB3610-PB08 (original locus, P*_dgcP_*-*dgcP*-*mV*-*yfp*), NCIB3610-PB85 (*amyE*::*P_xyl_*-*mV-yfp*-*dgcP*) and NCIB3610-PB86 (*amyE*::*P_xyl_*-*dgcP*-*mV-yfp*). (**B**) Comparison of *dgcP* gene fusion expression levels without xylose, expressed from its native locus and *amyE* loci respectively. (**C**) Comparative analysis of *mV*-*yfp*-*dgcP* and *dgcP*-*mV*-*yfp* expression levels by detection of the corresponding fusion protein using α-GFP antiserum upon addition of different inducer concentrations. Estimated size of all fusion proteins: 92 kDa as depicted by arrows. Asterisks indicate unspecific cross-reacting species.

**Table S1**. Strains used in this study

| **Strain** | **Relevant genotype** | **Source** |
| --- | --- | --- |

*B*. *subtilis* NCIB3610 Wild type, prototroph BGSC

DK1042 (NCIB3610) prototroph, *comI*^Q12L^ BGSC, 3A38

Gift from D. Kearns

Derivatives of NCIB3610 (DK1042, transformed with plasmid DNA)

NCIB3610-PB01 *P_dgcK_*-*dgcK*-*mV*-*yfp* (*cat*) This study

NCIB3610-PB08 *P_dgcP_*-*dgcP*-*mV*-*yfp* (*cat*) This study

NCIB3610-PB37 *amyE*::*P_xyl_*-*dgcK*-*cfp* (*spec*), This study

*comI*^Q12L^

NCIB3610-PB53 *P_xyl_*-*ydaJKLMN* (*cat*), *comI*^Q12L^ Bedrunka & Graumann,

2017

NCIB3610-PB55 *P_xyl_*-*ydaKLMN* (*cat*), *comI*^Q12L^ Bedrunka & Graumann,

2017

NCIB3610-PB56 *P_xyl_*-*ydaLMN* (*cat*), *comI*^Q12L^ Bedrunka & Graumann,

2017

NCIB3610-PB57 *amyE*::*P_xyl_*-*ydaK*-*mV*-*yfp* (*spec*), This study

*comI*^Q12L^

NCIB3610-PB79 *amyE*::*P_xyl_*-*mV*-*yfp*-*dgcK* (*spec*)*,* This study

*comI*^Q12L^

NCIB3610-PB85 *amyE*::*P_xyl_*-*mV*-*yfp*-*dgcP* (*spec*), This study

*comI*^Q12L^

NCIB3610-PB86 *amyE*::*P_xyl_*-*dgcP*-*mV*-*yfp* (*spec*), This study

*comI*^Q12L^

NCIB3610-PB87 *amyE*::*P_xyl_*-*mV*-*yfp*-*dgcW* (*spec*), This study

*comI*^Q12L^

NCIB3610-PB88 *amyE*::*P_xyl_*-*dgcW*-*mV*-*yfp* (*spec*), This study

*comI*^Q12L^

NCIB3610-PB90 *amyE*::*P_xyl_*-*dgcK*-*mV*-*yfp* (*spec*), This study

*comI*^Q12L^

NCIB3610-PB100 *amyE*::*P_xyl_*-*ydaKΔ4tmh*-*mV*-*yfp* This study

(*spec*), *comI*^Q12L^

NCIB3610-1193 *amyE*::*P_xyl_*-*mV*-*yfp* (*spec*), This study

*comI*^Q12L^

NCIB3610-XG001 *amyE*::*P_IPTG_*-*dgcW* (*spec*), This study

*comI*^Q12L^

NCIB3610-XG002 *amyE*::*P_IPTG_*-*dgcP* (*spec*), This study

*comI*^Q12L^

NCIB3610-XG003 *amyE*::*P_IPTG_*-*ydaK* (*spec*), This study

*comI*^Q12L^

NCIB3610-XG004 *amyE*::*P_IPTG_*-*dgcK* (*spec*), This study

*comI*^Q12L^

NCIB3610-XG086 *amyE*::*P_IPTG_*-*dgcW*Δ*eal* (*spec*), This study

*comI*^Q12L^

NCIB3610-PB37-PB10 *amyE*::*P_xyl_*-*dgcK*-*cfp* (*spec*)*,* pPB10 🡪

*ydaK*-*mV*-*yfp* (*cat*), *comI*^Q12L^ NCIB3610-PB37

This study

NCIB3610-PB56-PB57 *P_xyl_*-*ydaLMN* (*cat*), pPB57🡪

*amyE*::*P_xyl_*-*ydaK*-*mV*-*yfp* (*spec*), NCIB3610-PB56

*comI*^Q12L^ This study

**Table S1**. Strains used in this study

| **Strain** | **Relevant genotype** | **Source** |
| --- | --- | --- |

NCIB3610-PB56- *P_xyl_*-*ydaLMN* (*cat*), Bedrunka & Graumann,

pSG1193NLMV *amyE*::*P_xyl_*-*mV*-*yfp* (*spec*), 2017

*comI*^Q12L^

NCIB3610-PB56-PB80 *P_xyl_*-*ydaLMN* (*cat*), pPB80 🡪

*amyE*::*P_IPTG_*-*ydaK*^R202A^ (*spec*), NCIB3610-PB56

*comI*^Q12L^ This study

NCIB3610-PB56-PB81 *P_xyl_*-*ydaLMN* (*cat*), pPB81 🡪

*amyE*::*P_IPTG_*-*ydaK*^D205A^ (*spec*), NCIB3610-PB56

*comI*^Q12L^ This study

NCIB3610-PB56-PB100 *P_xyl_*-*ydaLMN* (*cat*), pPB100🡪

*amyE*::*P_xyl_*-*ydaKΔ4tmh*-*mV*-*yfp* NCIB3610-PB56

(*spec*), *comI*^Q12L^ This study

NCIB3610-PB56-XG003 *P_xyl_*-*ydaLMN* (*cat*), pXG003🡪 *amyE*::*P_IPTG_*-*ydaK* (*spec*), NCIB3610-PB56

*comI*^Q12L^ This study

Derivatives of NCIB3610 wild type (transformed with chromosomal DNA)

DS9305 Δ*dgcK* Gao *et al*., 2013

DS9305-PB55 Δ*dgcK*, *P_xyl_*-*ydaKLMN* (*cat*) NCIB3610-PB55

🡪DS9305

This study

DS9537 *dgcP*::*tet* Gao *et al*., 2013

DS9537-PB55 *dgcP*::*tet*, *P_xyl_*-*ydaKLMN* (*cat*) NCIB3610-PB55

🡪DS9537

This study

DS9883 Δ*dgcW* Gao *et al*., 2013

DS9883-PB55 Δ*dgcW*, *P_xyl_*-*ydaKLMN* (*cat*) NCIB3610-PB55

🡪DS9883

This study

DS1809 Δ*dgcK* Δ*dgcW* *dgcP*::*tet* Gift from D. Kearns Lab.

DS1809-PB53 Δ*dgcK* Δ*dgcW* *dgcP*::*tet*, NCIB3610-PB53

*P_xyl_*-*ydaJKLMN* (*cat*) 🡪DS1809

This study

DS1809-PB55 Δ*dgcK* Δ*dgcW* *dgcP*::*tet*, NCIB3610-PB55

*P_xyl_*-*ydaKLMN* (*cat*) 🡪DS1809

This study

DS1809-PB55-XG001 Δ*dgcK* Δ*dgcW* *dgcP*::*tet*, NCIB3610-XG001 *P_xyl_*-*ydaKLMN* (*cat*), 🡪DS1809-PB55

*amyE*::*P_IPTG_*-*dgcW* (*spec*) This study

DS1809-PB55-XG002 Δ*dgcK* Δ*dgcW* *dgcP*::*tet*, NCIB3610-XG002

*P_xyl_*-*ydaKLMN* (*cat*), 🡪DS1809-PB55

*amyE*::*P_IPTG_*-*dgcP* (*spec*) This study

DS1809-PB55-XG004 Δ*dgcK* Δ*dgcW* *dgcP*::*tet*, NCIB3610-XG004

*P_xyl_*-*ydaKLMN* (*cat*), 🡪DS1809-PB55

*amyE*::*P_IPTG_*-*dgcK* (*spec*) This study

DS1809-PB55-XG086 Δ*dgcK* Δ*dgcW* *dgcP*::*tet*, NCIB3610-XG086

*P_xyl_*-*ydaKLMN* (*cat*), 🡪DS1809-PB55

*amyE*::*P_IPTG_*-*dgcW*Δ*eal* (*spec*) This study

**Table S1**. Strains used in this study

| **Strain** | **Relevant genotype** | **Source** |
| --- | --- | --- |

DS1809-PB55-PB79 Δ*dgcK* Δ*dgcW* *dgcP*::*tet*, NCIB3610-PB79

*P_xyl_*-*ydaKLMN* (*cat*), 🡪DS1809-PB55

*amyE*::*P_xyl_*-*mV*-*yfp*-*dgcK* (*spec*) This study

DS1809-PB55-PB85 Δ*dgcK* Δ*dgcW* *dgcP*::*tet*, NCIB3610-PB85

*P_xyl_*-*ydaKLMN* (*cat*), 🡪DS1809-PB55

*amyE*::*P_xyl_*-*mV*-*yfp*-*dgcP* (*spec*) This study

DS1809-PB55-PB86 Δ*dgcK* Δ*dgcW* *dgcP*::*tet*, NCIB3610-PB86

*P_xyl_*-*ydaKLMN* (*cat*), 🡪DS1809-PB55

*amyE*::*P_xyl_*-*dgcP*-*mV*-*yfp* (*spec*) This study

DS1809-PB55-PB90 Δ*dgcK* Δ*dgcW* *dgcP*::*tet*, NCIB3610-PB90

*P_xyl_*-*ydaKLMN* (*cat*), 🡪DS1809-PB55

*amyE*::*P_xyl_*-*dgcK*-*mV*-*yfp* (*spec*) This study

DS1809-PB55- Δ*dgcK* Δ*dgcW* *dgcP*::*tet*, NCIB3610-1193

pSG1193NLMV *P_xyl_*-*ydaKLMN* (*cat*), 🡪DS1809-PB55

*amyE*::*P_xyl_*-*mV*-*yfp* (*spec*) This study

**Table S2**. Plasmids and vectors used in this study

| **Vector/ plasmid** | **Relevant genotype** | **Source** |
| --- | --- | --- |

pSG1164 *bla*, *cat*, *yfp*  Lewis, Martson, 1999

pSG1164-NLMV *bla*, *cat*, *mVyfp* Lab. stock

pSG1192 *bla*, *spec*, *cfp* Lewis, Martson, 1999

pSG1193-NLMV *bla*, *spec*, *mVyfp* Lab. stock

pSG1729-MVYFP *bla*, *spec*, *mVyfp* Lab. stock

pSG1164-NLMV-PB01 *P_dgcK_*-*dgcK*-*mV*-*yfp* (*cat*) This study

pSG1164-NLMV-PB08 *P_dgcP_*-*dgcP*-*mV*-*yfp* (*cat*) This study

pSG1164-NLMV-PB10 *P_ydaK_*-*ydaK*-*mV*-*yfp* (*cat*) Bedrunka & Graumann,

2017

pSG1192-CFP-PB37 *amyE*::*P_xyl_*-*dgcK*-*cfp* (*spec*) This study

pSG1164-PB53 *P_xyl_*-*ydaJKLMN* (*cat*) Bedrunka & Graumann,

2017

pSG1164-PB55 *P_xyl_*-*ydaKLMN* (*cat*) Bedrunka & Graumann,

2017

pSG1164-PB56 *P_xyl_*-*ydaLMN* (*cat*) Bedrunka & Graumann,

2017

pSG1193-NLMV-PB57 *amyE*::*P_xyl_*-*ydaK*-*mV*-*yfp* (*spec*) Bedrunka & Graumann,

2017

pSG1729-MVYFP-PB79 *amyE*::*P_xyl_*-*mV*-*yfp*-*dgcK* (*spec*) This study

pXG003 *amyE*::*P_IPTG_*-*ydaK* (*spec*) Gao *et al*., 2013

pXG003-PB80 *amyE*::*P_IPTG_*-*ydaK*^R202A^ (*spec*) This study

pXG003-PB81 *amyE*::*P_IPTG_*-*ydaK*^D205A^ (*spec*) This study

pSG1193-NLMV-PB90 *amyE*::*P_xyl_*-*dgcK*-*mV*-*yfp* (*spec*) This study

pSG1729-MVYFP-PB85 *amyE*::*P_xyl_*-*mV*-*yfp*-*dgcP* (*spec*) This study

pSG1193-NLMV-PB86 *amyE*::*P_xyl_*-*dgcP*-*mV*-*yfp* (*spec*) This study

pSG1729-MVYFP-PB87 *amyE*::*P_xyl_*-*mV*-*yfp*-*dgcW* (*spec*) This study

pSG1193-NLMV-PB88 *amyE*::*P_xyl_*-*dgcW*-*mV*-*yfp* (*spec*) This study

pSG1193-NLMV-PB100 *amyE*::*P_xyl_*-*ydaKΔ4tmh*-*mV*-*yfp* This study

(*spec*)

**Table S2**. Plasmids and vectors used in this study

| **Vector/ plasmid** | **Relevant genotype** | **Source** |
| --- | --- | --- |

pXG001 *amyE*::*P_IPTG_*-*dgcW* (*spec*) Gao *et al*., 2013

pXG002 *amyE*::*P_IPTG_*-*dgcP* (*spec*) Gao *et al*., 2013

pXG004 *amyE*::*P_IPTG_*-*dgcK* (*spec*) Gao *et al*., 2013

pXG086 *amyE*::*P_IPTG_*-*dgcW*Δ*eal* (*spec*) Gao *et al*., 2013

**Table S3**. Oligonucleotides used in this study

| **Primer** | **Sequence 5´- 3´** |
| --- | --- |

PB01f AAGGAGATTCCTAGGATGGGTACCGGAggcgtgtataaccgaagaaaat

PB01f CCTCCCAGGCCAGATAGGCCGGGCCCttcttttttttctgaaaaacacac

PB08f AAGGAGATTCCTAGGATGGGTACCGGAgctgtaaccaattcaatgcttc

PB08r CCTCCCAGGCCAGATAGGCCGGGCCCttttattgagtcatgaatcatcaa

PB16f CATGGGCCCATGAAAATATCATTCAGTG

PB16r ACGACTAGTTTATAGTTCATTCATCATC

PB19r TCGACTAGTTTATTCTGTATCTGTCTTTC

PB20f CATGGGCCCATGGTAGAACAAAC

PB21f CATGGGCCCTTGCTGAAAGAACTG

PB37r ACGGAATTCTTCTTTTTTTTCTG

PB57r CATGGGCCCTAGTTCATTCATCATC

PB79r CATGAATTCTCATTCTTTTTTTTCTGAAAAAC

PB80f GACAAGCGTTGCGGAAACGGATAAG

PB80r CTGATTTGCTGACCGACATATTG

PB81f GGGACGGAATTTCTTAGCCGTTTCCCGAACGCT

PB81r AGCGTTCGGGAAACGGCTAAGAAATTCCGTCCC

PB85r CATGAATTCTTATTTTATTGAGTCATG

PB86f CATGAATTCATGGTAGAACAAACTAAAG

PB86r CATGGGCCCTTTTATTGAGTCATGAATC

PB87f CATGGGCCCATGGGATTCGGTATTTGG

PB87r CATCTCGAGTTATTGCGACGGCTGTTC

PB88r CATGGTACCATGGGATTCGGTATTTG

PB88f CATGGGCCCTTGCGACGGCTGTTCAATAATG

PB90f CATGAATTCTTGCTGAAAGAACTGTTTG

PB90r CATGGGCCCTTCTTTTTTTTCTGAAAAAC

PB100f CATGAATTCATGCACGATATTACAGCAG

PB103f CATGAATTCATGATCATGAAACAAATG

PG5050f CAATTATTAGAGGTCATCGTTC

2928 *dgcW*f AGGAGGAATTCATCCTGCCAAAACAACGCCA

2931 *dgcW*r CTCCTGGATCCCAGGGTATAGGCCTTCGT

2932 *ydaK*f AGGAGGAATTCGTCTTTGAAGACATACAATATG

2935 *ydaK*r CTCCTGGATCCCTCTGAGCTGTTTCGCCAC

2936 *dgcK*f AGGAGGAATTCTCGTTGAAGTCGGTCTAGCTC

2939 *dgcK*r CTCCTGGATCCTCGGGATTGCTGAGTCTGAC

3037 *dgcP*f AGGAGGGATCCGTCTGCTCTATTCGACCATG

3040 *dgcP*r CTCCTCTCGAGCAGAAATTGTGCTTCCGATTC
